# Synthesis, biological evaluation and molecular docking analysis of vaniline–benzylidenehydrazine hybrids as potent tyrosinase inhibitors

**DOI:** 10.1186/s13065-020-00679-1

**Published:** 2020-04-07

**Authors:** Aida Iraji, Tina Adelpour, Najmeh Edraki, Mahsima Khoshneviszadeh, Ramin Miri, Mehdi Khoshneviszadeh

**Affiliations:** 1 Medicinal and Natural Products Chemistry Research Center, Shiraz University of Medical Sciences, Shiraz, Iran; 2 Department of Medicinal Chemistry, Faculty of Pharmacy, Shiraz University of Medical Sciences, Shiraz, Iran

**Keywords:** Anti-tyrosinase agents, Methoxybenzohydrazide, Molecular docking, Structure-based design, Kinetic study

## Abstract

In this work, 11 novel compounds based on vaniline and benzylidenehydrazine structure were synthesized with various substituents on phenyl aromatic ring of the molecule and evaluated as tyrosinase inhibitors. These new derivatives showed significant anti-tyrosinase activities, among which **4i** demonstrated to be the most potent compound, with IC_50_ values of 1.58 µM . The structure–activity relationship study of the novel constructed analogs was fully discussed. Kinetic study of compound **4i** showed uncompetitive inhibition towards tyrosinase. Furthermore, the high potency of **4i** was supported theoretically by molecular docking evaluations.
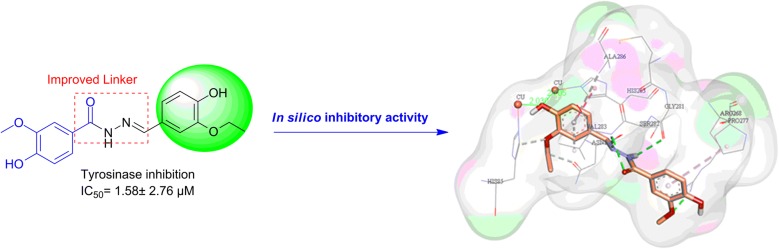

## Introduction

Melanin as the main dark macromolecular pigment is responsible for skin color and plays an important role in the protection of the skin against UV light induced damage [1]. However, hyperpigmentation disorders have been suggested to be involved in melasma, freckles, melanoma and responsible for serious and undesirable medicinal phenomena especially in elder people [2]. On the other hand, the major problem happened in food products including impairing the color, losing nutrition, texture and flavor. This unusual process results from the action of a group of enzymes especially tyrosinase (EC 1.14.18.1) as the rate-limiting enzyme which widely distributed in bacteria, fungi, plants and humans [3, 4]. The rate of enzymatic hyperpigmentation depends on the concentration of phenolic substrates, oxygen, reactive oxygen and nitrogen species, pH, temperature and tyrosinase [5]. In addition, tyrosinase inhibitors are becoming important in the cosmetic industry, due to their skin-whitening agents [6]. Furthermore, it has been reported that tyrosinase might contribute to the autoimmune disease [7], dopamine neurotoxicity and neurodegenerative disorder [8, 9]. These findings encouraged researchers to identify effective tyrosinase inhibitors for various applications in the food, cosmetics, and medicinal industries. Tyrosinase multifunctional copper-containing enzyme possesses both monophenolase and diphenolase activity which involved in catalyzing the hydroxylation of tyrosine (monophenolase activity) to 3,4-dihydroxyphenylalanine (o-diphenol or DOPA), and the oxidation of DOPA to dopaquinone (o-quinone) [10].

The literature survey reveals that a large number of tyrosinase inhibitors have been discovered from natural, synthetic and semi-synthetic sources including tropolone derivatives [11], hydroquinone derivatives, kojic acid [12], arbutin, bibenzyl glycosides [13]. Despite a large number of reported tyrosinase inhibitors, due to limitations of tyrosinase antagonism including safety and low efficacy, designing of new tyrosinase inhibitors is high demand [14, 15].

## Results and discussion

### Design of novel vaniline-benzylidenehydrazine hybrid

**Fig. 1:**
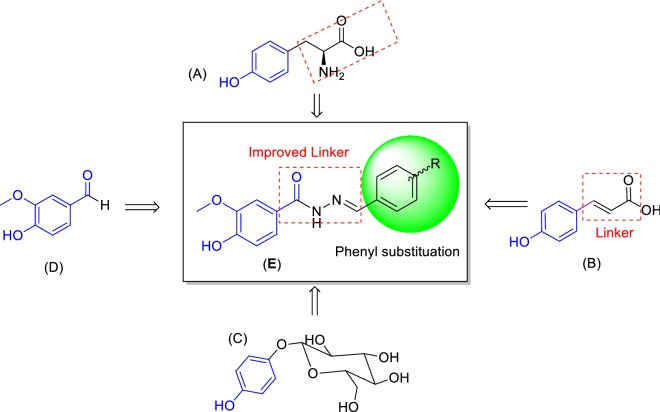
Design of new compounds targeting tyrosinase based on the hybridization strategies

Tyrosine (Tyr), (Fig. 1, **A**) is one of the natural ligands of tyrosinase and modified synthetic derivatives of tyrosine have been proved to exhibit potent anti-tyrosinase activity [14]. As a result, this skeleton was chosen for further modification. Most of the used tyrosinase inhibitors such as kojic acid, *p*-coumaric acid (Fig. 1, **B**) arbutine (Fig. 1, **C**) hydroxy-*N*ʹ-methylenebenzohydrazide [16] and oxyresveratrol containing free phenolic hydroxyl group [17]. The simple phenols such as hydroquinone, resorcin and its derivatives have been reported as potent phenolic inhibitors of the tyrosinase [1]. Phenols as small and natural compounds could be useful in the designing of more potent inhibitors. Vanillin as a natural product and flavoring agent is extensively used in food and cosmetics (Fig. 1, **D**) with consumption of around 200 tons each year [18].

The vanillin derivatives were reported to exhibit promising anti-tyrosinase activities, with the IC_50_ values of 16.13 µM for the most potent compound. Besides, the molecular docking study confirmed the role of phenols motif in H-bound interaction with Ser206 residue.

- As part of our efforts in identifying tyrosinase candidate inhibitor, we became interested in hybrids obtained by linking of vanillin and some fragments of l-tyrosine.
- As a result molecule **E** was designed to explore the structural requirements of tyrosinase-inhibitory activity including the one methoxyl on *meta* and one hydroxy group on *para* position of the phenyl ring based on vanillin structure and previously reported inhibitor. Structural modifications were performed on linker type and length of l-tyrosine and *p*-coumaric acid. As a result, acyl hydrazide was supposedly aimed at structurally stability, reducing the probability of cleavage, the capability to involve in hydrogen bond interaction and expanding the opportunity for further derivatization. The number of reported aromatic aldehydes possessing anti-tyrosinase activity containing electron-donating or electron-withdrawing groups on 3 or 4 positions of benzene ring [19] has been selected for their potential inhibition of tyrosinase.

### Chemistry

**Scheme 1:**
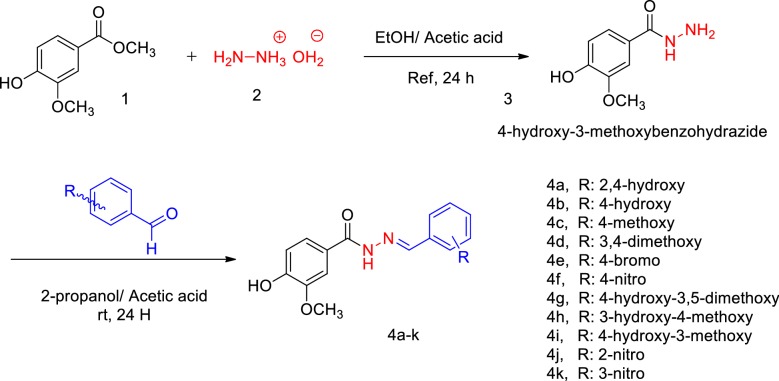
Synthesis of (E)-*N*ʹ-benzylidene-4-hydroxy-3-methoxybenzohydrazide derivatives **4a**–**k**

The general synthetic routes for preparing the target compounds are illustrated in Scheme 1.

**Fig. 2:**
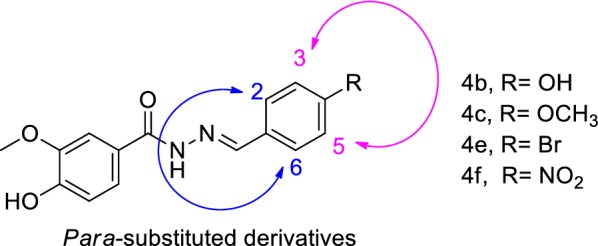
*Para*-substituted derivatives of benzylidene-4-hydroxy-3-methoxybenzohydrazide

The 4-hydroxy-3-methoxybenzohydrazide intermediate (**3**, Scheme 1) were prepared using commercially available methyl 4-hydroxy-3-methoxybenzoate (**1**, Scheme 1) and hydrazine hydrate (**2**, Scheme 1) in refluxing ethanol in the presence of catalytic amounts of acetic acid under simple nucleophilic substitution reaction and completion of the reaction was monitored by TLC to give compound [20]. The treatment of **3** with an appropriate aldehyde in the presence of acetic acid in 2-propanol yielded the desired final products **4a**–**k** (80–92%) [21]. Structural determination and signal assignments of the final compounds were accomplished by the application of IR, MASS and NMR experiments. ^1^H and ^13^C spectra are available in Additional file 1. The peaks that caught our particular attention were ^13^C spectra of **4b** (*para*-substituted derivatives) in which 13 carbon signal was expected to be seen, since 15 signals were observed. The same pattern were observed for other *para*-substituted compounds (**4c**, **4e** and **4f**). There is evidence of the hydrazine motif presence that the two *ortho* ring carbons (Fig. 2. carbons 2 and 6, compound **4b, 4c** and **4e)** and the two *meta* ring carbons (carbons 3 and 5 compound **4b**, **4c** and **4e**) are magnetically inequivalent (although equivalent by symmetry) and each gives different ^13^C single peak. Similar evidence was reported in previous studies containing hydrazine linker [20, 22].

### Tyrosinase inhibitory activity

The series of vaniline-benzylidenehydrazine conjugates underwent biological screening for their inhibitory potential against tyrosinase. All data of 11 benzylidenehydrazine-tyrosinase derivatives and kojic acid as reference compounds were summarized in Table 1.

**Table 1:** Biological evaluation of the inhibitory activity of (E)-*N*ʹ-benzylidene-4-hydroxy-3-methoxybenzohydrazide derivatives and kojic acid against tyrosinase enzyme in the presence of L-DOPA as the substrate
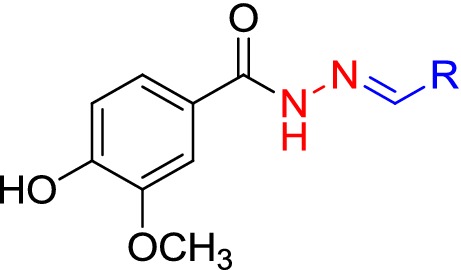

Compounds	R	IC_50_ (µM)^a^	Compounds	R	IC_50_ (µM) ^a^
**4a**		37.09 ± 1.02	**4g**		4.58 ± 1.23
**4b**		15.21 ± 1.34	**4h**		10.00 ± 1.6
**4c**		35.63 ± 2.8	**4i**		1.58 ± 2.76
**4d**		20.95 ± 2.09	**4j**		17.01 ± 2.03
**4e**		1.95 ± 2.34	**4k**		5.84 ± 1.9
**4f**		29.06 ± 0.89	**Kojic acid**^b^		9.3 ± 1.27

^a^Data presented here are the mean ± SEM of three to five independent experiments

^b^Reference compound

A survey on the IC_50_ values against the tyrosinase enzyme revealed that all compounds had significant inhibitory activity at micro-molar levels with IC_50_ values ranging from 1.58 to 37.09 μM. To find the influence of the type of substitution of the aryl ring on the potency of compounds the structure–activity analyses have been performed.

As it is obvious in Table 1, the aromatic ring substituents had a significant effect on tyrosinase inhibition.

- Enzymatic assays revealed that compounds **4i** and **4e** exhibited the best activities against tyrosinase enzyme with IC_50_ values of 1.58 and 1.95 μM respectively, surpassing that of the positive control kojic acid (IC_50_  =  9.3 μM).
- The comparison of nitro-substituted compounds **4f**, **4j** and **4k** demonstrated that the introduction of the nitro group resulted in the improvement of inhibitory activity. The activity of these analogs changes in the following order: *meta *> *orto* > *para*. Moreover, high potency of the compounds with the strong electron-withdrawing substituent for inhibition of tyrosinase enzyme was also observed.
- In the case of compounds containing only OH substituent on the benzene ring, moderate inhibitory activity was observed. In this respect, compounds **4a** possessing 2, 4-OH substitutions (IC_50_ =  37.09 ± 1.02 μM) and **4b** with 4-OH substitutions (IC_50_  =  15.21 ± 1.34 μM) on the benzene ring demonstrated moderate potencies. Surprisingly, adding extra methoxy group with electron-donating properties on the benzene ring can increase the inhibitory activity significantly like **4h** with IC_50_ of 10.00 µM and **4** **g** with IC_50_ of 4.58 µM.
- Analyzing the isosteric substitution of a hydroxyl group with OMe substituent (**4c**, **4d**, **4g** and **4h**) showed the desired IC_50_ value. The low inhibitory activity was observed in compound **4c** with one OMe groups while **4g** with two OMe groups in 3,5 positions and one OH on 4 position was revealed higher potency (IC_50_ =  4.58 ± 1.23 μM). The effects of the OMe group depending on their position, bulkiness as well as the presence of the potential hydrogen bond.
- The effect of the length of the etheric moiety of the benzene aromatic ring was also studied. Accordingly, replacing the methoxy group with ethoxy chain **4i** did improve the inhibitory potency significantly (IC_50_ =  1.58 ± 2.76 μM) which was around sixfold more potent than reference drug kojic acid.
- Biological evaluation of motifs at the *para*-position of the phenyl ring suggesting the importance of the hydrophobic character for the interaction with the enzyme. Compound **4e** with the Br substituent on the *para*-position exhibits the promising activity with the IC_50_ values of 1.95 μM. Compounds **4b** and **4c** with OH or OMe group in the same position are slightly weaker inhibitors.

### Kinetic study of tyrosinase inhibition

**Fig. 3:**
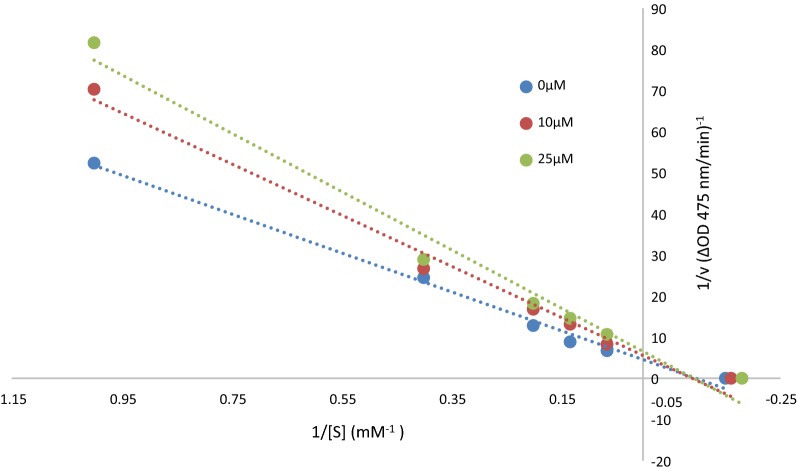
Lineweaver–Burk plot for the inhibition of tyrosinase-catalyzed l-DOPA oxidation by **4i** at 0, 10 and 25 µM

To gain further insight into the mechanism of action of this family of tyrosine-like compounds, a kinetic study was carried out with the most promising inhibitors of tyrosinase enzyme. Lineweaver–Burk plot was obtained for **4i** the most potent inhibitor with L-DOPA as the substrate the data summarized in Table 2 and Fig. 3. The inhibition constants, K_i_, and V_m_ for tyrosinase inhibitors were determined by fitting the kinetic data to a competitive, noncompetitive, or mixed inhibition model by nonlinear regression analysis using GraphPad Prism. As shown in Fig. 3, with increasing concentrations of compound **4i**, the K_m_ and V_m_ decreased. Therefore, this compound is a uncompetitive inhibitor for the tyrosinase enzyme.

**Table 2:** Kinetic parameters for the compounds **4i** against mushroom tyrosinase inhibition assay

Compound	K_m_ (mM)	V_m_ (OD/Min)
**4i** (0 µM)	61.489	1.68
**4i** (10 µM)	11.18	0.18
**4i** (25 µM)	12.08	0.17

### Molecular docking analysis

Tyrosinase enzyme structure contains two H subunits with 392 residues and two L subunits with 150 residues. The H subunit of tyrosinase contains a binuclear copper site in which three histidine residues name His61, His85 and His94 interact with first copper ion and the second Cu ion coordinated with His259, His263, and His296. The most important factor involved in tyrosinase inhibition is trapping histidine amino acid coordinated with Cu ion which played an important role in the activity.

In order to gain insight into the interactions and binding mode of the synthesized compounds in the active site of tyrosinase enzyme, the molecular docking analysis was performed. The 3D coordinate of the tyrosinase (PDB ID: 2Y9X) in complex with tropolone was retrieved from Protein Data Bank (PDB) at http://www.rcsb.org/pdb/home/home.do. Before docking targeted protein and synthetic derivatives were prepared and the best pose with the lowest binding energy was selected for further analysis. Docking validation was done by extracting the structure of the co-crystallized ligand and re-docking it into the receptor (self-docking) with an RMSD of 2.49 Å.

**Fig. 4:**
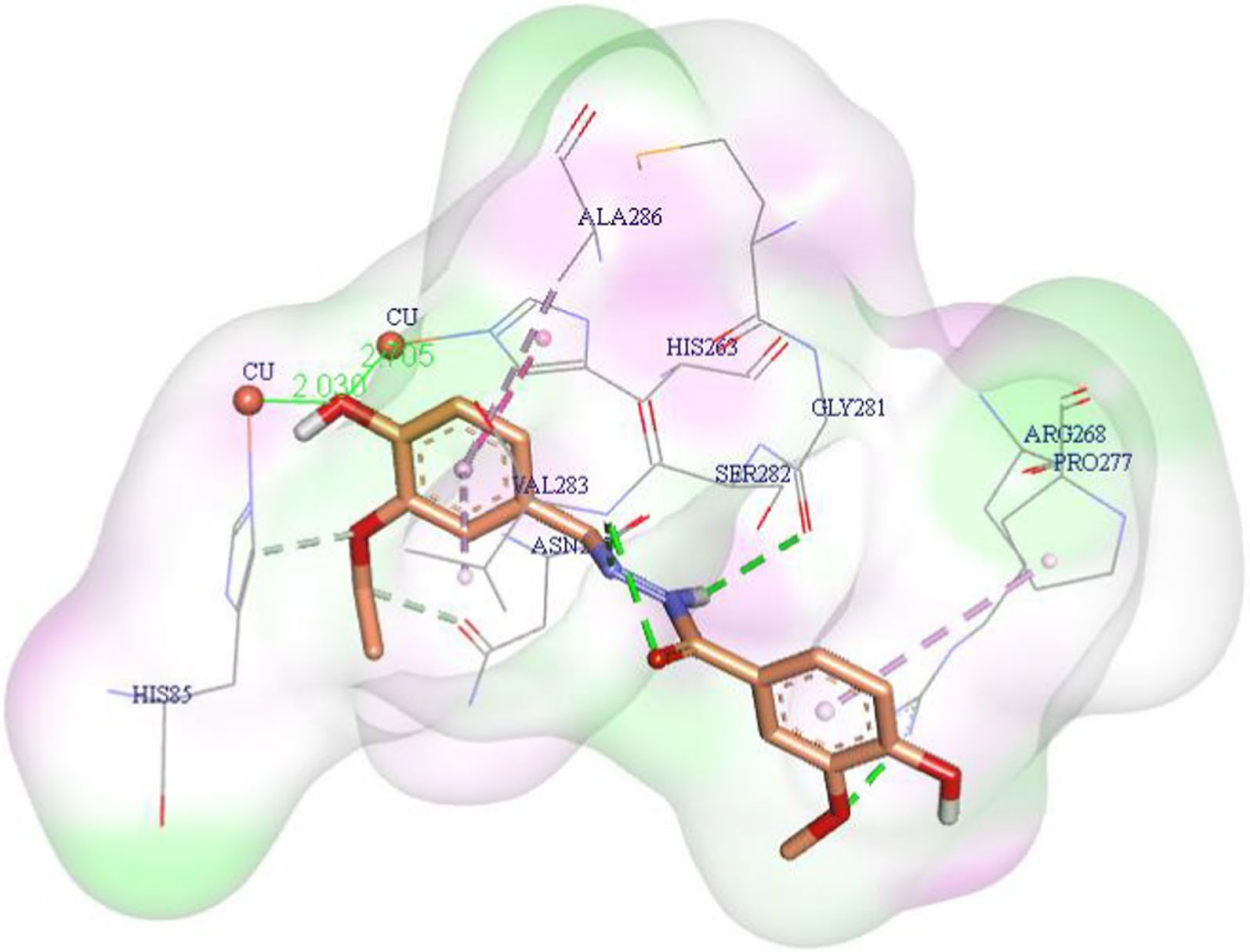
The binding mode of compound of **4i** within mushroom tyrosinase (PDB ID: 2Y9X) active site. **4i** is presented in orange. Only important residues for binding are shown. Hydrogen bonds interactions were depicted in green, van der Waals interaction were depicted in green, interaction with metal ions were depicted in the green line, π–π and π–aryl stacking interactions were depicted in pink

The compound **4i** showed excellent anti-tyrosinase activity with IC_50_ of 1.58  µM, due to the presence of O–CH_2_–CH_3_ as a bulky electron-donating group on the 3rd position and OH on the 4th position of the phenyl ring. This orientation of the **4i** enables it to make a hydrogen bond of ethoxy moiety with His85 (Fig. 4). In this position, the 3-ethoxy-4-hydroxybenzylidene ring was well fitted in the active site through π-aryl interaction with Ala286 and Val283. The 4-OH of hydroxy benzylidene ring from two interactions with the imidazole group of His85 (2.03 Å) and His263 (2.7 Å). NH of hydrazine linker was involved in H-bond interactions with CO of the backbone of Gly281 (distance 2.70 Å) while the Nʹ atom of hydrazine linker formed H-bond interaction with Val283 (distance: 3.01 Å). The carbonyl moieties of **4i** created additional hydrogen bond interaction with Val283 (distance: 2.23 Å). On the other side of the ligand, the plain structure of benzyl moiety contributes to making a staking interaction with Pro277. In this situation, the methoxy group binds to Arg268 through H-bound interaction. Detailed molecular docking studies confirmed the importance of hydrophobic and hydrogen bonds interactions between **4i** as inhibitor and tyrosinase.

## Conclusion

The presence of phenolic structural features may help in the designing of more potent tyrosinase inhibitors. Novel 11 vaniline-benzylidenehydrazine hybrids were investigated as tyrosinase inhibitor. The results showed that four compounds have high tyrosinase inhibitory activities with IC_50_ values below positive control. Among them, **4i** derivative showed the best antityrosinase activity (IC_50_  =  1.58 ± 2.76 µM), 6 times better in comparison with kojic acid (IC_50_  =  9.3 ± 1.27 µM) followed by **4e** with IC_50_ of 1.95. Moreover, it is worth mentioning that **4i** showed a competitive inhibition mode of action. Inspection of the chemical structures, it can be concluded that the tyrosinase inhibitory activity was related to the substituent groups at C3 and C4 positions of benzohydrazide moiety. The free OH group at *para* positions of the benzyl ring increased the hydrophilicity and H-bound interaction capability in this region which fulfills the minimum structural features of novel designed compounds. The SAR study by the modification of substituent revealed that optimum bulkiness at *meta* positions of the benzyl ring can also improve the inhibition potential of compounds. Docking simulation showed that the **4i** illustrated a lot of interactions with the active site of tyrosinase. And the potential may be due to the formation of strong interactions with His85, His263 through copper ion and hydrogen bonds interaction with Arg268, Gly281 and Val283 as well as π-aryl and Van der Waals interactions with the amino acid residues located inside the active site.

The straightforward synthesis and high potency of newly designed tyrosine-like compounds **4a**–**k** introduce them as attractive lead compounds towards the discovery of effective tyrosinase inhibitors.

## Material and method

### Chemistry

All reagents were reagent grade quality and obtained from Sigma-Aldrich (Prague, Czech Republic). The reaction process was monitored using thin layer chromatography on the glass-backed silica gel sheets (Silica Gel 60 GF254) and visualized under UV light (254 nm). Column chromatography was performed on silica gel (90–150 mm; Merck Chemical Inc.). ^1^H and ^13^C NMR spectra were determined by a Bruker FT-300 MHz spectrometer in DMSO-d_6_. All the chemical shifts were reported as (δ) values (ppm). Mass spectra were obtained on Agilent 7890A spectrometer at 70 eV. The infrared (IR) spectra were run as KBr disk on Perki-Elmer Spectrum RXI FTIR.

#### Procedure for the synthesis of methyl 4-hydroxy-3-methoxybenzoate (**3**)

Methyl 4-hydroxy-3-methoxybenzoate (**1**, 10 mmol) and hydrazine hydrate (**2**, 30 mmol) were added to 100 mL EtOH in the presence of catalytic amount of acetic acid. The mixture was refluxed for 24 h. The filtered residue was purified by recrystallization in ethanol. The residues was then washed three times with 5 mL cold ethanol. Finally, the solid was dried in a vacuum at 50  °C to give 3 without further purification. White solid, 93% yield. Melting point: 135.0 °C

#### General procedure for the synthesis of compounds **4a**–**k**

Methyl 4-hydroxy-3-methoxybenzoate (**3**, 2 mmol) was then added into 20 mL 2-propanol as a solvent. To the resulting solution different selected aldehyde (2.2 mmol) were added. On completion of reaction (TLC) the precipitate were filtered and recrystallized from ethanol. Subsequently dried under reduced pressure to provide the (**4a**–**i**) product.

##### Synthesis of (E)-*N*ʹ-(2,4-dihydroxybenzylidene)-4-hydroxy-3-methoxybenzohydrazide (**4a**)

Compound **4a** was prepared by the described procedure to afford pure yellow crystalline solid. Yield: 79%; Melting point: 243.0 °C; FT-IR (KBr): υ = 3524, 3420, 3164, 2875, 1640, 1613, 1259; ^1^HNMR (300 MHz, DMSO-*d*_*6*_, ppm): *δ*_H_ 11.42 (s, 1H, hydrazine-N–H), 11.37 (s, 1H, 2,4-dihydroxybenzylidene-N=CH), 11.06 (s, 1H, 2,4-dihydroxybenzylidene-OH), 10.18 (s, 1H, 2,4-dihydroxybenzylidene-OH), 9.93 (s, 1H, 4-hydroxy-3-methoxy benzohydrazide-OH), 8.19 (m, 2H, 4-hydroxy-3-methoxybenzohydrazide-C_2,6_-H), 8.13 (s, 1H, 4-hydroxy-3-methoxy benzohydrazide-C_5_-H), 7.39 (d, *J *= 6.0 Hz, 1H, 2,4-dihydroxybenzylidene-C_3_-H), 7.26 (d, *J *= 6.0 Hz, 2H, 2,4-dihydroxybenzylidene-C_5,6_-H), 3.83 (s, 3H, 4-hydroxy-3-methoxy benzohydrazide-OCH_3_); ^13^C NMR (DMSO *d*_*6*_, 125 MHz): *δ*_c_ (ppm) = 163.9, 159.7, 150.4, 147.7, 129.2, 125.9, 124.8, 121.6, 121.3, 120.0, 116.2, 115.4, 111.9, 109.7, 56.2; MS (EI) *m/z* (%): 302 (M^+,^ 5), 194 (40), 151 (30), 135 (100), 123 (18), 60 (35), 43 (30).

##### Synthesis of (E)-4-hydroxy-*N*ʹ-(4-hydroxybenzylidene)-3-methoxybenzohydrazide (**4b**)

Golden powder; Yield: 75%; Melting point: 256 °C; FT-IR (KBr): υ = 3521, 3405, 3024, 2870, 1646, 1605, 1269; ^1^HNMR (300 MHz, DMSO-*d*_6_, ppm): *δ*_H_ 11.45 (s, 1H, hydrazine-N-H), 9.92 (s, 1H, 4-hydroxybenzylidene-N=CH), 9.71 (s, 1H, 4-hydroxybenzylidene-OH), 8.34 (s, 1H, 4-hydroxy-3-methoxy benzohydrazide-OH), 7.48 (m, 2H, 4-hydroxybenzylidene-C_2,6_-H), 7.44 (m, 2H, 4-hydroxy-3-methoxy-C_2,6_-H), 6.86 (m, 3H, 4-hydroxy-3-methoxy benzohydrazide-C_5_-H and 4-hydroxybenzylidene-C_3,5_-H), 3.85 (s, 3H, 4-hydroxy-3-methoxy benzohydrazide-OCH_3_); ^13^C NMR (DMSO *d*_*6*_, 125 MHz): *δ*_c_ (ppm) = 163.9, 159.7, 150.4, 147.7, 129.2, 125.9, 124.8, 121.6, 121.3, 120.0, 116.2, 115.4, 111.9, 109.7, 56.2; MS (EI) *m/z* (%): 286 (M^+^, 10), 167 (40), 151 (100), 136 (11), 123 (20), 108 (10), 77 (112).

##### Synthesis of (E)-4-hydroxy-3-methoxy-*Nʹ*-(4-methoxybenzylidene)benzohydrazide (**4c**)

White powder; Yield: 76%; Melting point: 110.0 °C; FT-IR (KBr): υ = 3523, 3393, 3016, 2841, 1509, 1364, 1209. ^1^HNMR (300 MHz, DMSO-*d*_*6*_, ppm): *δ*_H_ 11.55 (s, 1H, hydrazine-N–H), 9.76 (s, 1H, 4-methoxybenzylidene-N=CH), 8.40 (s, 1H, 4-hydroxy-3-methoxy benzohydrazide-OH), 7.67 (s, 1H, 4-hydroxy-3-methoxy benzohydrazide-C_2_-H), 7.50 (brs, 1H, 4-hydroxy-3-methoxy benzohydrazide-C_6_-H), 7.46 (d, *J *= 6.0 Hz, 2H, 4-methoxybenzylidene-C_2,6_-H), 7.01 (brs, 1H, 4-hydroxy-3-methoxy benzohydrazide-C_5_-H), 6.89 (d, *J *= 6.0 Hz, 2H, 4-methoxybenzylidene-C_3,5_-H), 3.85 (s, 3H, 4-hydroxy-3-methoxybenzohydrazide-OCH_3_), 3.80 (s, 3H, 4-methoxybenzylidene-OCH_3_); ^13^CNMR (DMSO-*d*_*6*_, 125 MHz): *δ*c (ppm) = 163.1, 161.1, 150.5, 149.7, 148.4, 138.3, 129.0, 127.5, 124.7, 121.7, 118.4, 115.4, 114.8, 112.0, 56.1, 55.7; MS (EI) *m/z* (%): 300 (M^+^, 50), 268 (10), 168 (85), 151 (100), 123 (15), 105 (12), 77 (10).

##### Synthesis of (E)-*N*ʹ-(3,4-dimethoxybenzylidene)-4-hydroxy-3-methoxy benzohydrazide (**4d**)

White powder; Yield: 75%; Melting point: 231.0 °C; FT-IR (KBr): υ = 3313, 3009, 2964, 2838, 1639, 1597, 1364; ^1^HNMR (300 MHz, DMSO-*d*_6_, ppm): *δ*_H_ 11.58 (s, 1H, hydrazine-N–H), 9.74 (s, 1H, dimethoxybenzylidene-N=CH), 8.39 (s, 1H, 4-hydroxy-3-methoxy benzohydrazide-OH), 7.50 (s, 1H, 3,4-dimethoxybenzylidene-C_2_-H), 7.39 (s, 1H, 4-hydroxy-3-methoxy benzohydrazide-C_2_-H), 7.18 (brs, 1H, 4-hydroxy-3-methoxy benzohydrazide-C_6_-H), 7.02 (m, 2H, 3,4-dimethoxybenzylidene-C_6_-H and 4-hydroxy-3-methoxy benzohydrazide-C_5_-H), 6.89 (d, *J *= 6.0 Hz, 1H, 3,4-dimethoxybenzylidene-C_5_-H), 3.85 (s, 3H, 4-hydroxy-3-methoxy benzohydrazide-OCH_3_), 3.81 (s, 6H, 3,4-dimethoxybenzylidene-2(OCH_3_)); ^13^C NMR (DMSO-*d*_*6*_, 125 MHz): *δ*_c_ (ppm) = 163.1, 151.7, 150.5, 149.5, 147.7, 127.6, 124.7, 122.2, 122.0, 121.7, 115.4, 112.0, 111.9, 108.58, 56.2, 56.0, 55.9; MS (EI) *m/z* (%): 330 (M^+^, 18), 207 (5), 180 (20), 167 (40), 151 (100), 123 (20), 77 (10), 65 (12).

##### Synthesis of (E)-*N*ʹ-(4-bromobenzylidene)-4-hydroxy-3-methoxybenzohydrazide (**4e**)

Yellow powder; Yield: 91%; Melting point: 115.0 °C; FT-IR (KBr): υ = 3331, 3046, 2929, 2839, 1654, 1593, 1438; ^1^HNMR (300 MHz, DMSO-*d*_6_,ppm): *δ*_H_ 12.46 (s, 1H, hydrazine-N–H), 11.85 (s, 1H, 4-bromobenzylidene-N=CH), 8.43 (s, 1H, 4-hydroxy-3-methoxy benzohydrazide-OH), 7.90 (s, 1H, 4-hydroxy-3-methoxy benzohydrazide-C_2_-H), 7.68 (m, 4H, 4-bromobenzylidene C_2_,_3,5,6_-H), 6.53 (brs, 2H, 4-hydroxy-3-methoxy benzohydrazide-C_5,6_-H), 3.80 (s, 3H, 4-hydroxy-3-methoxy benzohydrazide-OCH_3_); ^13^C NMR (DMSO-*d*_*6*_, 125 MHz): *δ*_c_ (ppm) = 164.8, 163.4, 161.7, 150.0, 149.9, 146.5, 132.9, 131.3, 129.0, 128.5, 122.9, 106.9, 105.9, 100.8, 54.9; MS (EI) *m/z* (%): 348 (M^+2,^ 6), 348 (M, 6), 167 (40), 151 (100), 123 (20), 89 (12).

##### Synthesis of (E)-4-hydroxy-3-methoxy-*N*ʹ-(4-nitrobenzylidene)benzohydrazide (**4f**)

Yellow powder; Yield: 81%; Melting point: 195.0 °C; FT-IR (KBr): υ = 3093, 2970, 2927, 2840, 1643, 1621, 1523, 1477; ^1^HNMR (300 MHz, DMSO-*d*_6_, ppm): *δ*_H_ 12.22 (s, 1H, hydrazine-N-H), 11.98 (s, 1H, 4-nitrobenzylidene-N=CH), 8.55 (s, 1H, 4-hydroxy-3-methoxy benzohydrazide-OH), 8.30 (s, 1H, 4-hydroxy-3-methoxy benzohydrazide-C_2_-H), 7.99 (m, 2H, 4-nitrobenzylidene-C_3,5_-H), 7.91 (m, 2H, 4-nitrobenzylidene-C_2,6_-H), 6.57 (m, 2H, 4-hydroxy-3-methoxy-benzohydrazide-C_5,6_-H), 3.80 (s, 3H, 4-hydroxy-3-methoxy benzohydrazide-OCH_3_); ^13^C NMR (DMSO-*d*_*6*_, 125 MHz): *δ*_c_ (ppm) = 163.5, 163.5, 162.5, 161.5, 147.4, 145.1, 144.9, 140.0, 129.3, 127.5, 123.5, 106.9, 106.0, 101.0, 54.9; MS (EI) *m/z* (%): 315 (M^+,^ 10), 167 (20), 151 (100), 135 (10), 123 (18), 108 (18), 59 (24).

##### Synthesis of (E)-4-hydroxy-*N*ʹ-(4-hydroxy-3,5-dimethoxybenzylidene)-3-methoxy benzohydrazide (**4g**)

White powder; Yield: 85%, Melting point:151 °C; FT-IR (KBr): υ = 3521, 3434, 3227, 2965, 1643, 1558, 1426; ^1^HNMR (300 MHz, DMSO-*d*_6_, ppm): *δ*_H_ 11.52 (s, 1H, hydrazine-N–H), 9.73 (s, 1H, 4-hydroxy-3,5-dimethoxybenzylidene-N=CH), 8.91 (s, 1H, 4-hydroxy-3-methoxy benzohydrazide-OH), 8.34 (s, 1H, 4-hydroxy-3,5-dimethoxybenzylidene-OH), 7.46 (s, 1H, 4-hydroxy-3-methoxybenzohydrazide–C_2_–H), 7.44 (s, 1H, 4-hydroxy-3-methoxybenzohydrazide–C_6_–H), 6.89 (brs, 2H, 4-hydroxy-3,5-dimethoxybenzylidene –C_2_,_6_–H), 6.87 (brs, 1H, 4-hydroxy-3-methoxybenzohydrazide–C_5_–H), 3.85 (s, 3H, 4-hydroxy-3-methoxy benzohydrazide-OCH_3_), 3.82 (s, 6H, 4-hydroxy-3,5-dimethoxybenzylidene-2(OCH_3_)); ^13^C NMR (DMSO-*d*_*6*_, 125 MHz): *δ*_c_ (ppm) = 163.0, 157.7, 150.5, 148.6, 148.2, 147.7, 138.3, 125.2, 124.8, 121.7, 115.4, 112.1, 112.0, 104.9, 56.5, 56.4, 56.2; MS (EI) *m/z* (%): 346 (M^+,^ 15), 196 (10), 179 (15), 167 (30), 151 (100), 135 (10), 123 (20), 108 (10).

##### Synthesis of (E)-4-hydroxy-*N*ʹ-(3-hydroxy-4-methoxybenzylidene)-3-methoxybenzohydrazide (**4h**)

White powder; Yield: 90%; Melting point: 98.0 °C; FT-IR (KBr): υ = 3246, 3087, 3227, 2838, 1598, 1356, 1212; ^1^HNMR (300 MHz, DMSO-*d*_6_, ppm): *δ*_H_ 11.49 (s, 1H, hydrazine-N–H), 9.74 (s, 1H, 3-hydroxy-4-methoxybenzylidene-N=CH), 9.35 (s, 1H, OH), 8.30 (s, 1H, 4-hydroxy-3-methoxy benzohydrazide-OH), 7.49 (s, 1H, 4-hydroxy-3-methoxy benzohydrazide-C_2_–H), 7.45 (d, *J *= 6.0 Hz, 1H, 4-hydroxy-3-methoxy benzohydrazide-C_6_–H), 7.28 (s, 1H, 3-hydroxy-4-methoxybenzylidene-C_2_–H), 7.05 (d, *J *= 6.0 Hz, 1H, 4-hydroxy-3-methoxy benzohydrazide-C_5_–H), 6.97 (d, *J *= 6.0 Hz, 1H, 3-hydroxy-4-methoxybenzylidene-C_6_–H), 6.88 (d, *J *= 6.0 Hz, 1H, 3-hydroxy-4-methoxybenzylidene-C_5_–H), 3.85 (brs, 3H, 3-hydroxy-4-methoxybenzylidene-OCH_3_), 3.81 (brs, 3H, 4-hydroxy-3-methoxy benzohydrazide-OCH_3_); ^13^C NMR (DMSO-*d*_*6*_, 125 MHz): *δ*_c_ (ppm) = 163.0, 150.5, 150.1, 147.7, 147.6, 147.3, 127.8, 124.8, 121.7, 120.6, 115.4, 112.7, 112.3, 112.0, 56.2, 56.0; MS (EI) *m/z* (%): 316 (M^+,^ 12), 180 (10), 167 (40), 151 (100), 134 (15), 123 (20), 106 (10).

##### Synthesis of (E)-*N*ʹ-(3-ethoxy-4-hydroxybenzylidene)-4-hydroxy-3-methoxybenzohydrazide (**4i**)

White powder; Yield: 76%; Melting point: 114.0 °C; FT-IR (KBr): υ = 3424, 3301, 3080, 2975, 1504, 1371, 1271; ^1^HNMR (300 MHz DMSO-*d*_6_, ppm): *δ*_H_ 12.63 (s, 1H, hydrazine-N–H), 11.65 (s, 1H, 3-ethoxy-4-hydroxybenzylidene-N=CH), 9.55 (s, 1H, 3-ethoxy-4-hydroxybenzylidene-OH), 8.34 (s, 1H, 4-hydroxy-3-methoxy benzohydrazide-OH), 7.91 (m, 2H, 4-hydroxy-3-methoxy benzohydrazide–C_2_–H and 3-ethoxy-4-hydroxybenzylidene-C_2_–H), 7.31 (brs, 1H, 4-hydroxy-3-methoxy benzohydrazide–C_6_–H), 7.11 (brs, 1H, 3-ethoxy-4-hydroxybenzylidene-C_6_–H), 6.87 (s, 1H, 3-ethoxy-4-hydroxybenzylidene-C_5_–H), 6.51 (brs, 1H, 3-ethoxy-4-hydroxybenzylidene-C_5_–H), 4.07 (q, *J *= 6 Hz, 2H, 3-ethoxy-4-hydroxybenzylidene-OCH_2_CH_3_), 3.80 (s, 3H, 4-hydroxy-3-methoxy benzohydrazide-OCH_3_), 1.37 (t, *J *= 6.0 Hz, 3H, 3-ethoxy-4-hydroxybenzylidene-OCH_2_CH_3_); ^13^C NMR (DMSO-*d*_*6*_, 125 MHz): δ_c_ (ppm) = 164.6, 163.3, 161.8, 148.9, 146.6, 128.7, 124.9, 121.7, 115.0, 109.7, 106.8, 105.8, 100.7, 100.4, 63.3, 55.5, 54.9; MS (EI) *m/z* (%): 328 (M^+^, 15), 222 (10), 207 (5), 163 (35), 151 (25), 135 (100), 123 (10), 105 (15).

##### Synthesis of (E)-4-hydroxy-3-methoxy-*N*ʹ-(2-nitrobenzylidene)benzohydrazide (**4j**)

White powder; Yield: 78%, Melting point: 151 °C; FT-IR (KBr): υ = 3093, 2970, 2970, 2840, 1643, 1630, 1523, 1477; ^1^HNMR (300 MHz DMSO-*d*_6_, ppm): *δ*_H_ 12.22 (s, 1H, hydrazine-N–H), 11.98 (s, 1H, 2-nitrobenzylidene-N=CH), 8.55 (s, 1H, 4-hydroxy-3-methoxy benzohydrazide-OH), 8.31 (s, 1H, 4-hydroxy-3-methoxy benzohydrazide–C_2_–H), 7.91 (m, 4H, 2-nitrobenzylidene–C_3_,_4,5,6_–H), 6.51 (brs, 2H, 4-hydroxy-3-methoxy benzohydrazide–C_5,6_–H), 3.81 (s, 3H, 4-hydroxy-3-methoxy benzohydrazide-OCH_3_); ^13^C NMR (DMSO-*d*_*6*_, 125 MHz): *δ*_c_ (ppm) = 164.8, 163.5, 162.5, 161.5, 147.4, 145.1, 144.9, 139.9, 129.3, 127.5, 123.5, 106.9, 105.9, 100.8, 54.9; MS (EI) *m/z* (%): 315 (M^+,^ 15), 167 (10), 151 (100), 135 (10), 107 (10), 95 (8).

##### Synthesis of (E)-4-hydroxy-3-methoxy-*N*ʹ-(3-nitrobenzylidene)benzohydrazide (**4k**)

White powder; Yield: 88%, Melting point:151 °C; FT-IR (KBr): υ = 3091, 2970, 2970, 2840, 1643, 1630, 1523, 1477; ^1^HNMR (300 MHz DMSO-*d*_6_, ppm): *δ*_H_ 11.62 (s, 1H, hydrazine-N–H), 11.47 (s, 1H, 3-nitro benzylidene-N=CH), 8.48 (s, 1H, 4-hydroxy-3-methoxy benzohydrazide-OH), 8.42 (s, 1H, 3-nitrobenzylidene–C_2_–H), 8.22 (m, 2H, 4-hydroxy-3-methoxy benzohydrazide–C_2_–H and 3-nitrobenzylidene–C_4_–H), 8.08 (m, 2H, 3-nitrobenzylidene–C_5,6_–H), 7.71 (m, 2H, 4-hydroxy-3-methoxy benzohydrazide-C_5,6_-H), 3.37 (s, 3H, 4-hydroxy-3-methoxy benzohydrazide-OCH_3_); ^13^C NMR (DMSO-*d*_*6*_, 125 MHz): *δ*_c_ (ppm) = 171.6, 165.4, 147.6, 142.6, 139.6, 135.6, 132.6, 132.1, 129.8, 123.5, 123.3, 120.3, 120.1, 21.1, 19.7; MS (EI) *m/z* (%):315 (M^+^, 15), 167 (10), 151 (100), 135 (10), 107 (10), 95 (8).

### Mushroom tyrosinase inhibition assay

All test samples were first dissolved in DMSO at 50 mM and diluted to the required concentrations. First, 10 ml of tyrosinase (0.5 mg ml) was mixed with 160 μl of 50 mM phosphate buffer (pH = 6.8) in 96-well microplates and then 10 μl of different concentration of the test sample was added. After 20 min incubation at 28 °C, 20 ml of L-Dopa solution (0.5 mM) was added to the phosphate buffer and the enzymatic activity was monitored by observing dopa quinone formation at 475 nm. DMSO without test compounds and kojic acid were used as the control and positive control respectively. The tyrosinase activity without inhibitor was defined as 100%. Each concentration was analyzed in three independent experiments run in triplicate. The inhibitory activity of the tested compounds was expressed as the concentration that inhibited 50% of the enzyme activity (IC_50_).

### Determining the inhibition type

To determine the inhibition kinetics of vaniline-benzylidenehydrazine a series of experiments were performed. Different concentrations of the **4i** (0, 10 and 25 µM) was chosen to get a series of straight lines. Pre-incubation and measurement time were the same as discussed in mushroom tyrosinase inhibition assay protocol. The maximal velocity (Vmax) and the Michaelis constant (K_m_) of the tyrosinase activity were determined by the Line weaver Burk plot at various concentrations of L-DOPA (0.25, 0.5, 0.75 and 1 mM) as a substrate. The inhibition type of the enzyme was assayed by Line weaver Burk plots of inverse of velocities (1/V) versus inverse of substrate concentrations 1/[S] mM.

### Molecular docking study

The X-ray crystal structure of tyrosinase (PDB code: 2Y9X) containing tropolone as the innate ligand in the binding site were obtained from protein data bank (http://www.rcsb.org). Water molecules and cognate ligand were excluded from 2Y9X, hydrogens were added, nonpolar hydrogens were merged and Gasteiger charges were calculated for protein. 3D structures of ligands were sketched and optimized (by molecular mechanics, MM^+^ and AM1, methods) using Hyperchem software. The PDBQT files were created by adding charges and defining the degree of torsions. The three-dimensional grids 60 * 60 * 60 (x, y, z) were created with a grid spacing of 0.375 Å and the cubic grids were centered on the binding site of native ligand comprise copper metal ions [23]. Lamarckian genetic algorithm (LGA) was applied to model the interaction/binding between then ligand and the tyrosinase active site. For Lamarckian GA, 27,000. The other parameters were left at program default values. The final binding mode described in the manuscript was selected taking into account the best-ranked scoring functions.

## Supplementary information

**Additional file 1: Figure S1.** Mass spectra of compound 4a. **Figure S2.**^1^H-NMR of compound 4a. **Figure S3.**^13^C-NMR of compound 4a. **Figure S4.** IR of compound 4a. **Figure S5.** Mass spectra of compound 4b. **Figure S6.**^1^H-NMR of compound 4b. **Figure S7.**^13^C-NMR of compound 4b. **Figure S8.** IR of compound 4b. **Figure S9.** Mass spectra of compound 4c. **Figure S10.**^1^H-NMR of compound 4c. **Figure S11.**^13^C-NMR of compound 4c. **Figure S12.** IR of compound 4c. **Figure S13.** Mass spectra of compound 4d. **Figure S14.**^1^H-NMR of compound 4d. **Figure S14.**^1^H-NMR of compound 4d. **Figure S16.** IR of compound 4d. **Figure S17.** Mass spectra of compound 4e. **Figure S18.**^1^H-NMR of compound 4e. **Figure S19.**^13^C-NMR of compound 4e. **Figure S20.** IR of compound 4e. **Figure S21.** Mass spectra of compound 4f. **Figure S22.**^1^H-NMR of compound 4f. **Figure S23.**^13^C-NMR of compound 4f. **Figure S24.** IR of compound 4f. **Figure S25.** Mass spectra of compound 4g. **Figure S26.**^1^H-NMR of compound 4g. **Figure S27.**^13^C NMR of compound 4g. **Figure S28.** IR of compound 4g. **Figure S29.** Mass spectra of compound 4h. **Figure S30.**^1^H-NMR of compound 4h. **Figure S31.**^13^C-NMR of compound 4h. **Figure S32.** IR of compound 4h. **Figure S33.** Mass spectra of compound 4i. **Figure S34.**^1^H-NMR of compound 4i. **Figure S35.**^13^C-NMR of compound 4i. **Figure S36.** IR of compound 4i. **Figure S37.** Mass of compound 4j. **Figure S38.**^13^H-NMR of compound 4j. **Figure S39.**^13^C-NMR of compound 4j. **Figure S40.** IR of compound 4j. **Figure S41.** Mass of compound 4k. **Figure S42.**^1^H-NMR of compound 4k. **Figure S43.**^13^C-NMR of compound 4k. **Figure S44.** IR of compound 4k.

## Data Availability

The datasets used and analyzed during the current study are available from the corresponding author on reasonable request. We have presented all data in the form of tables and figures.

## Abbreviations

L-Tyr: l-tyrosinase
L-DOPA: l-dihydroxyphenylalanine
Val: Valine
Gly: Glycine
Glu: Glutamic acid
Ala: Alanine
Asn: Asparagine
Arg: Arginine
His: Histidine
LGA: Lamarckian genetic algorithm
DMSO-d_6_: Deuterated dimethyl sulfoxide
^1^H NMR: Proton nuclear magnetic resonance
^13^C NMR: Carbon-13 nuclear magnetic resonance

## References

[1] Zolghadri S, Bahrami A, Hassan Khan MT, Munoz-Munoz J, Garcia-Molina F, Garcia-Canovas F, Saboury AA (2019). A comprehensive review on tyrosinase inhibitors. J Enzyme Inhib Med Chem.

[2] Baxter LL, Pavan WJ (2013). The etiology and molecular genetics of human pigmentation disorders. Wiley Interdiscip Rev Dev Biol.

[3] Mayer AM (2006). Polyphenol oxidases in plants and fungi: going places? A review. Phytochemistry.

[4] Halaouli S, Asther M, Sigoillot JC, Hamdi M, Lomascolo A (2006). Fungal tyrosinases: new prospects in molecular characteristics, bioengineering and biotechnological applications. J Appl Microbiol.

[5] Zheng Z-P, Cheng K-W, Chao J, Wu J, Wang M (2008). Tyrosinase inhibitors from paper mulberry (*Broussonetia papyrifera*). Food Chem.

[6] Oyama T, Takahashi S, Yoshimori A, Yamamoto T, Sato A, Kamiya T, Abe H, Abe T, Tanuma S-i (2016). Discovery of a new type of scaffold for the creation of novel tyrosinase inhibitors. Bioorg Med Chem.

[7] Lavezzo MM, Sakata VM, Morita C, Rodriguez EEC, Abdallah SF, da Silva FTG, Hirata CE, Yamamoto JH (2016). Vogt-Koyanagi-Harada disease: review of a rare autoimmune disease targeting antigens of melanocytes. Orphanet J Rare Dis.

[8] Adetumbi MA, Lau BH (1983). Alliumsativum (garlic)—a natural antibiotic. Med Hypotheses.

[9] Asanuma M, Miyazaki I, Ogawa N (2003). Dopamine-or L-DOPA-induced neurotoxicity: the role of dopamine quinone formation and tyrosinase in a model of Parkinson’s disease. Neurotox Res.

[10] Iraji A, Khoshneviszadeh M, Bakhshizadeh P, Edraki N (2019). Structure-based design, synthesis, biological evaluation and molecular docking study of 4-hydroxy-*N*ʹ-methylenebenzohydrazide derivatives acting as tyrosinase inhibitors as potentiate anti-melanogenesis activities. Med Chem.

[11] Liu J, Cao R, Yi W, Ma C, Wan Y, Zhou B, Ma L, Song H (2009). A class of potent tyrosinase inhibitors: alkylidenethiosemicarbazide compounds. Eur J Med Chem.

[12] Garcia A, Fulton JE (1996). The combination of glycolic acid and hydroquinone or kojic acid for the treatment of melasma and related conditions. Dermatol Surg.

[13] Tajima R, Oozeki H, Muraoka S, Tanaka S, Motegi Y, Nihei H, Yamada Y, Masuoka N, Nihei K-I (2011). Synthesis and evaluation of bibenzyl glycosides as potent tyrosinase inhibitors. Eur J Med Chem.

[14] Lee SY, Baek N, Nam T-G (2016). Natural, semisynthetic and synthetic tyrosinase inhibitors. J Enzyme Inhib Med Chem.

[15] Chang T-S (2009). An updated review of tyrosinase inhibitors. Int J Mol Sci.

[16] Aida I, Mahsima K, Pegah B, Najmeh E, Mehdi K (2019). Structure-based design, synthesis, biological evaluation and molecular docking study of 4-hydroxy-*N*ʹ-methylenebenzohydrazide derivatives acting as tyrosinase inhibitors as potentiate anti-melanogenesis activities. Med Chem.

[17] Rescigno A, Sollai F, Pisu B, Rinaldi A, Sanjust E (2002). Tyrosinase inhibition: general and applied aspects. J Enzyme Inhib Med Chem.

[18] Walton NJ, Mayer MJ, Narbad A (2003). Vanillin. Phytochemistry.

[19] Jiménez M, Chazarra S, Escribano J, Cabanes J, García-Carmona F (2001). Competitive inhibition of mushroom tyrosinase by 4-substituted benzaldehydes. J Agric Food Chem.

[20] Iraji A, Firuzi O, Khoshneviszadeh M, Nadri H, Edraki N, Miri R (2018). Synthesis and structure–activity relationship study of multi-target triazine derivatives as innovative candidates for treatment of Alzheimer’s disease. Bioorg Chem.

[21] Yazdani M, Edraki N, Badri R, Khoshneviszadeh M, Iraji A, Firuzi O (2019). Multi-target inhibitors against Alzheimer disease derived from 3-hydrazinyl 1,2,4-triazine scaffold containing pendant phenoxy methyl-1,2,3-triazole: design, synthesis and biological evaluation. Bioorg Chem.

[22] Khoshneviszadeh M, Ghahremani MH, Foroumadi A, Miri R, Firuzi O, Madadkar-Sobhani A, Edraki N, Parsa M, Shafiee A (2013). Design, synthesis and biological evaluation of novel anti-cytokine 1,2,4-triazine derivatives. Bioorg Med Chem.

[23] Edraki N, Iraji A, Firuzi O, Fattahi Y, Mahdavi M, Foroumadi A, Khoshneviszadeh M, Shafiee A, Miri R (2016). 2-Imino 2H-chromene and 2-(phenylimino) 2H-chromene 3-aryl carboxamide derivatives as novel cytotoxic agents: synthesis, biological assay, and molecular docking study. J Iran Chem Soc.

