# Supplementary material for: Synthesis, biological evaluation and molecular docking analysis of vaniline–benzylidenehydrazine hybrids as potent tyrosinase inhibitors
Source: BMC Chem. 2020 Apr 7;14(1):28. doi: 10.1186/s13065-020-00679-1 (PMC7137441; doi:10.1186/s13065-020-00679-1)
Supplement: Supplementary file 1 — Additional file 1: Figure S1. Mass spectra of compound 4a. Figure S2.1H-NMR of compound 4a. Figure S3.13C-NMR of compound 4a. Figure S4. IR of compound 4a. Figure S5. Mass spectra of compound 4b. Figure S6.1H-NMR of compound 4b. Figure S7.13C-NMR of compound 4b. Figure S8. IR of compound 4b. Figure S9. Mass spectra of compound 4c. Figure S10.1H-NMR of compound 4c. Figure S11.13C-NMR of compound 4c. Figure S12. IR of compound 4c. Figure S13. Mass spectra of compound 4d. Figure S14.1H-NMR of compound 4d. Figure S14.1H-NMR of compound 4d. Figure S16. IR of compound 4d. Figure S17. Mass spectra of compound 4e. Figure S18.1H-NMR of compound 4e. Figure S19.13C-NMR of compound 4e. Figure S20. IR of compound 4e. Figure S21. Mass spectra of compound 4f. Figure S22.1H-NMR of compound 4f. Figure S23.13C-NMR of compound 4f. Figure S24. IR of compound 4f. Figure S25. Mass spectra of compound 4g. Figure S26.1H-NMR of compound 4g. Figure S27.13C NMR of compound 4g. Figure S28. IR of compound 4g. Figure S29. Mass spectra of compound 4h. Figure S30.1H-NMR of compound 4h. Figure S31.13C-NMR of compound 4h. Figure S32. IR of compound 4h. Figure S33. Mass spectra of compound 4i. Figure S34.1H-NMR of compound 4i. Figure S35.13C-NMR of compound 4i. Figure S36. IR of compound 4i. Figure S37. Mass of compound 4j. Figure S38.13H-NMR of compound 4j. Figure S39.13C-NMR of compound 4j. Figure S40. IR of compound 4j. Figure S41. Mass of compound 4k. Figure S42.1H-NMR of compound 4k. Figure S43.13C-NMR of compound 4k. Figure S44. IR of compound 4k. [file 13065_2020_679_MOESM1_ESM.docx]

**Figure S1. Mass spectra of compound 4a**


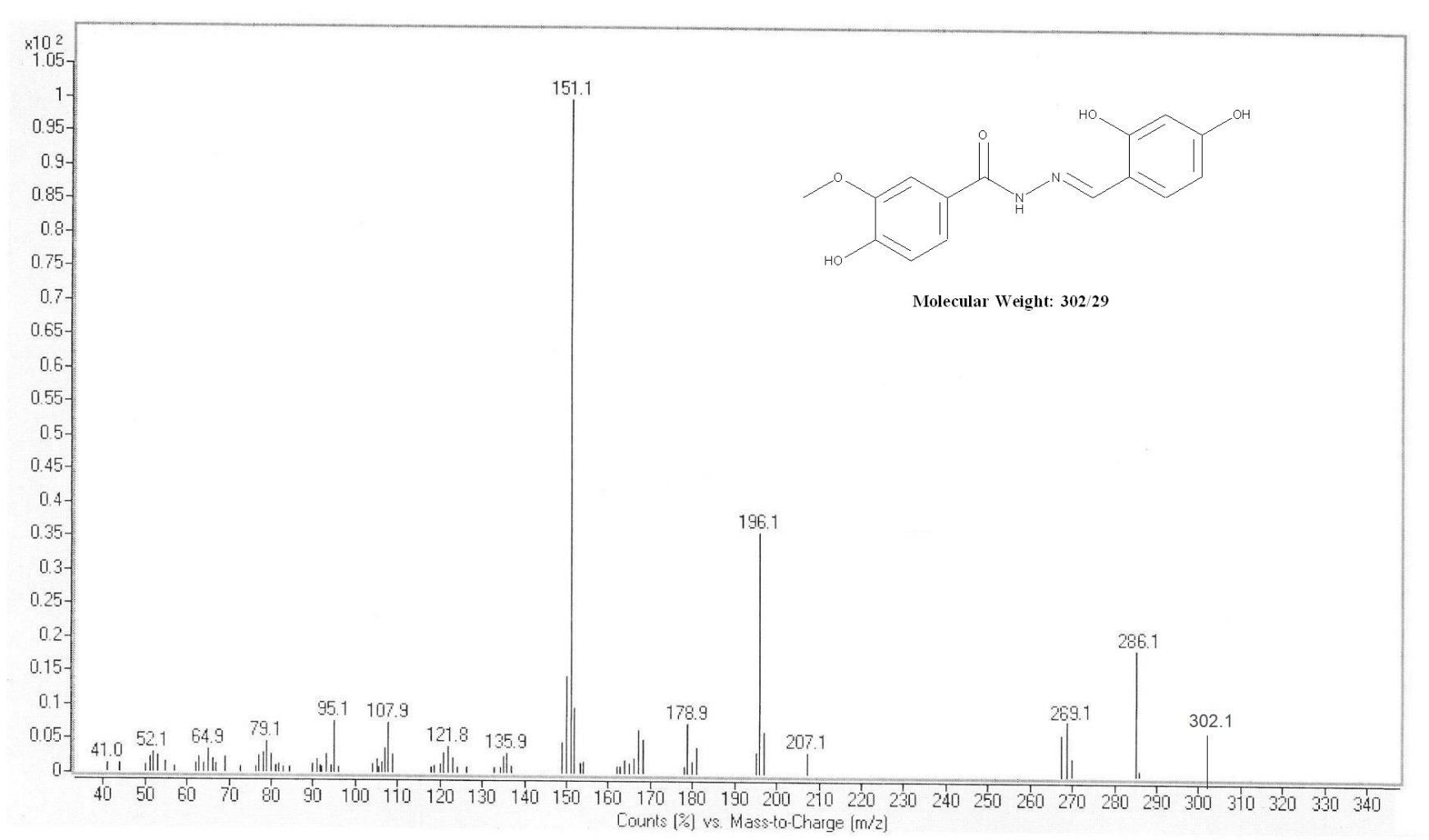


**Figure S2. ^1^H-NMR of compound 4a**


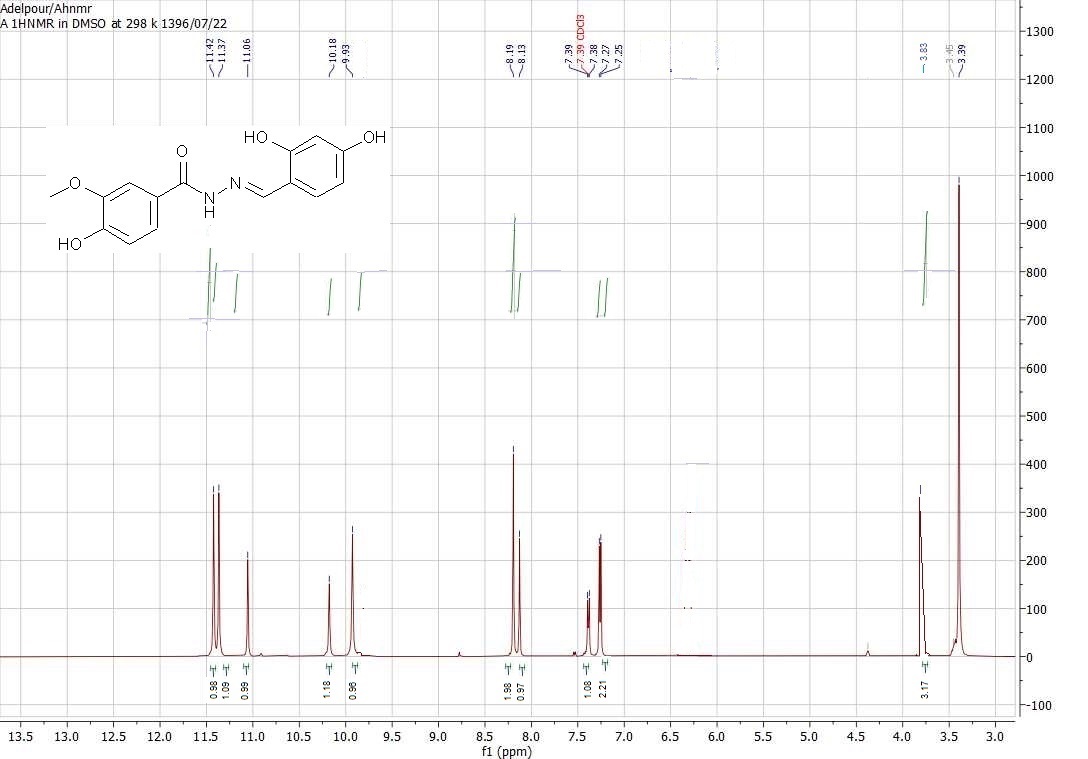


**Figure S3. ^13^C-NMR of compound 4a**


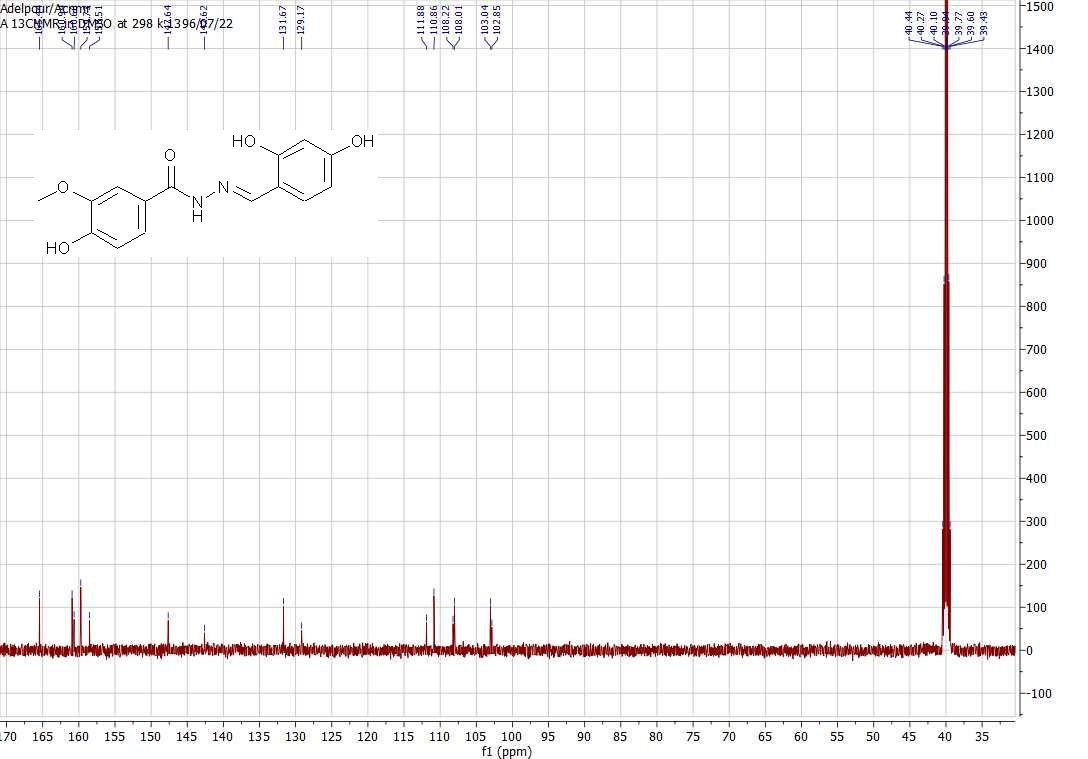


**Figure S4. IR of compound 4a**


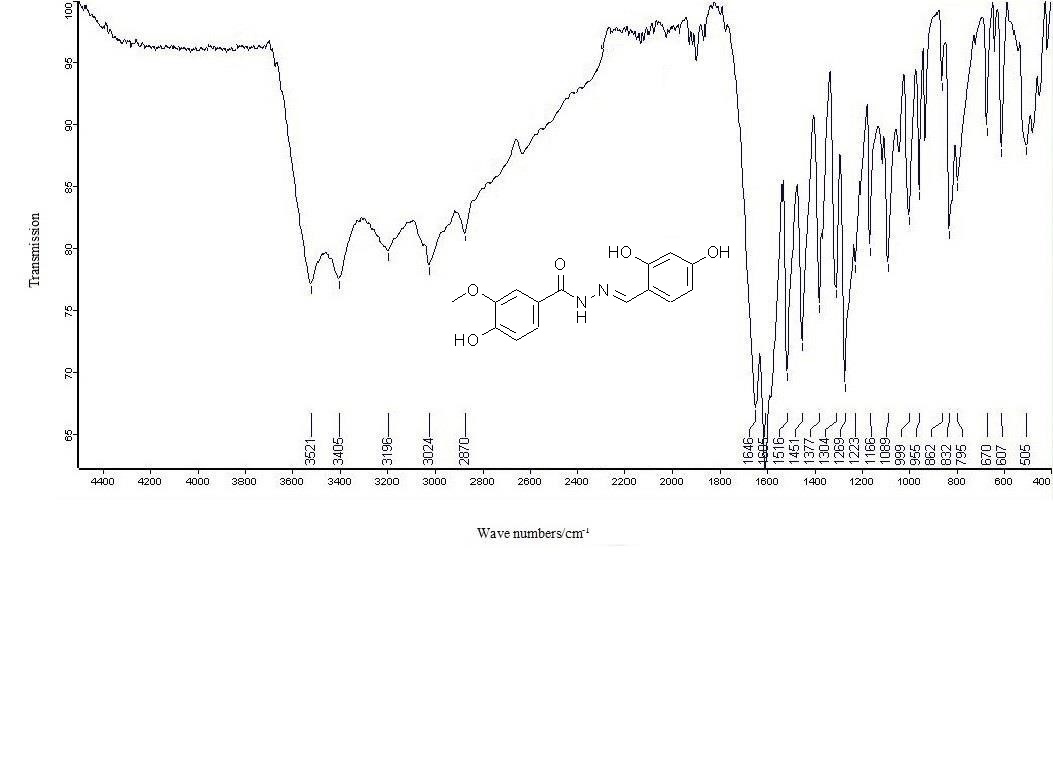


**Figure S5. Mass spectra of compound 4b**

**
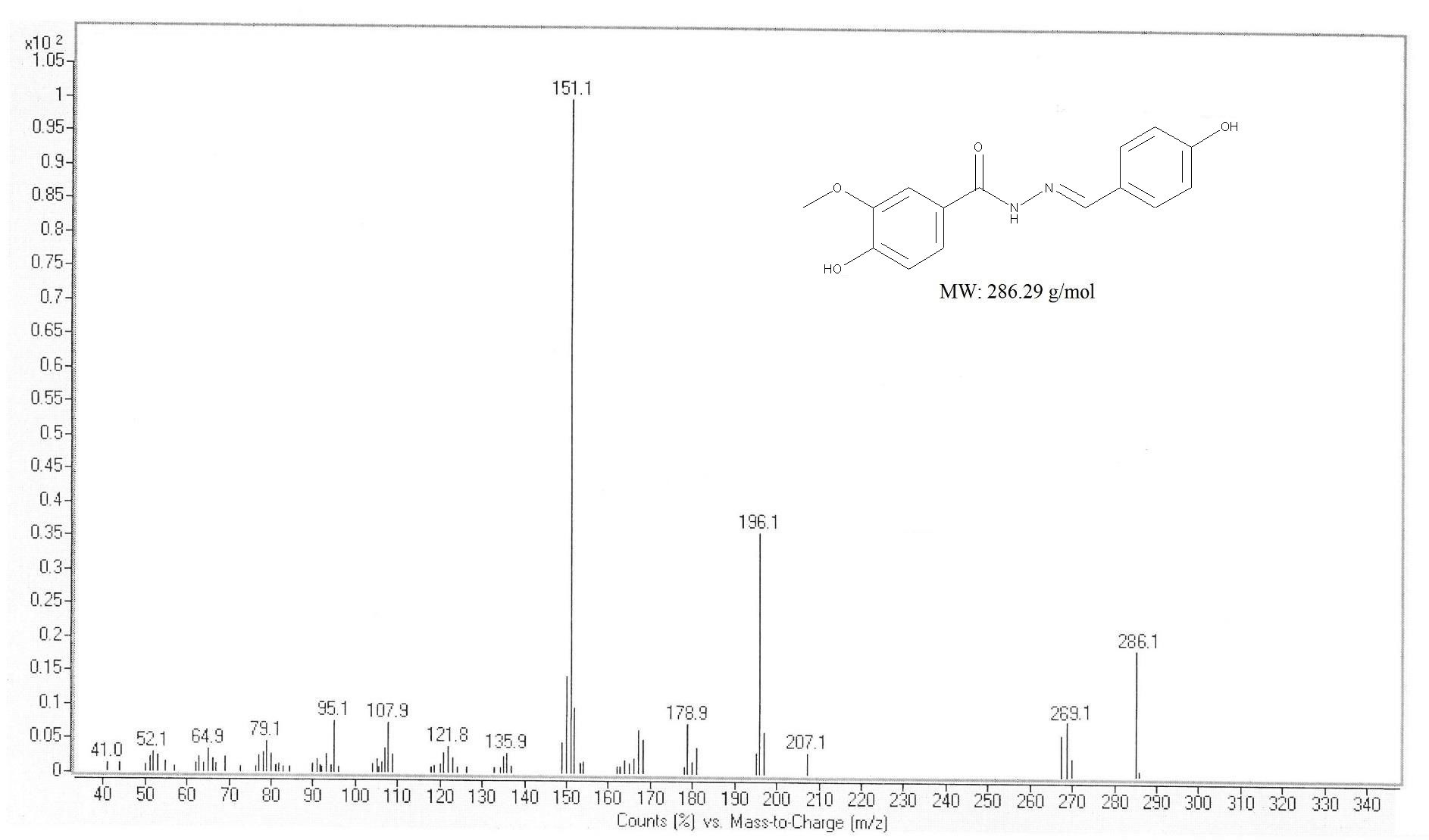
**

**Figure S6. ^1^H-NMR of compound 4b**

**
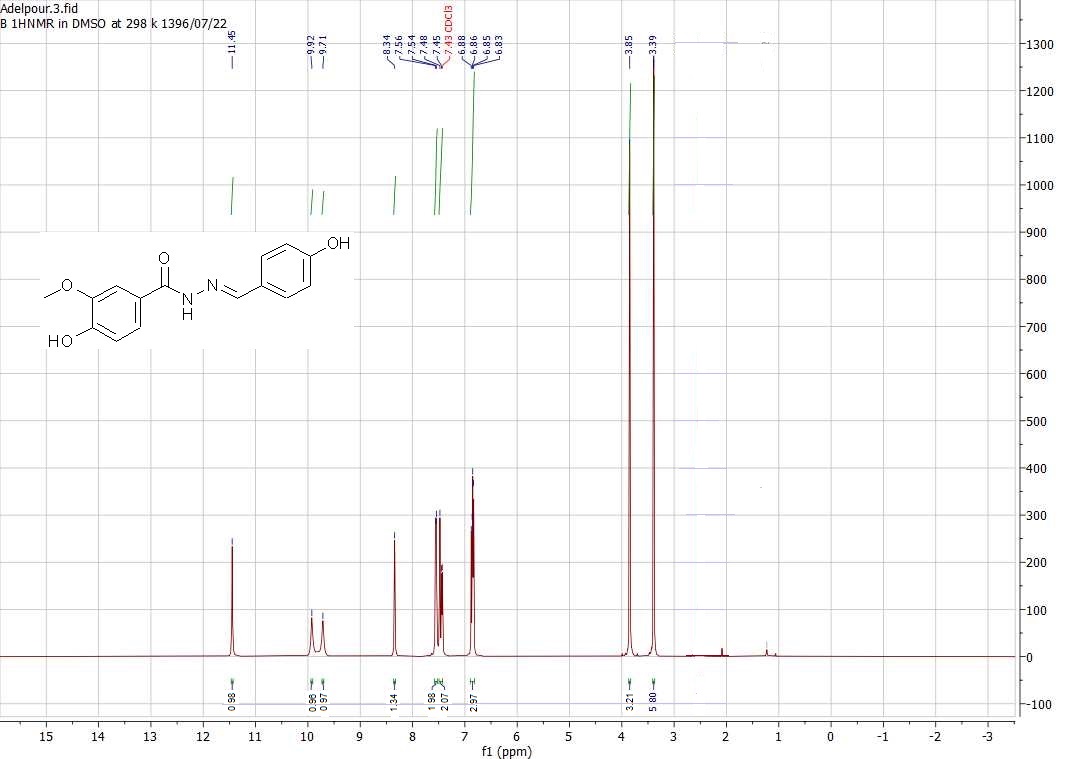
**

**Figure S7. ^13^C-NMR of compound 4b**

**
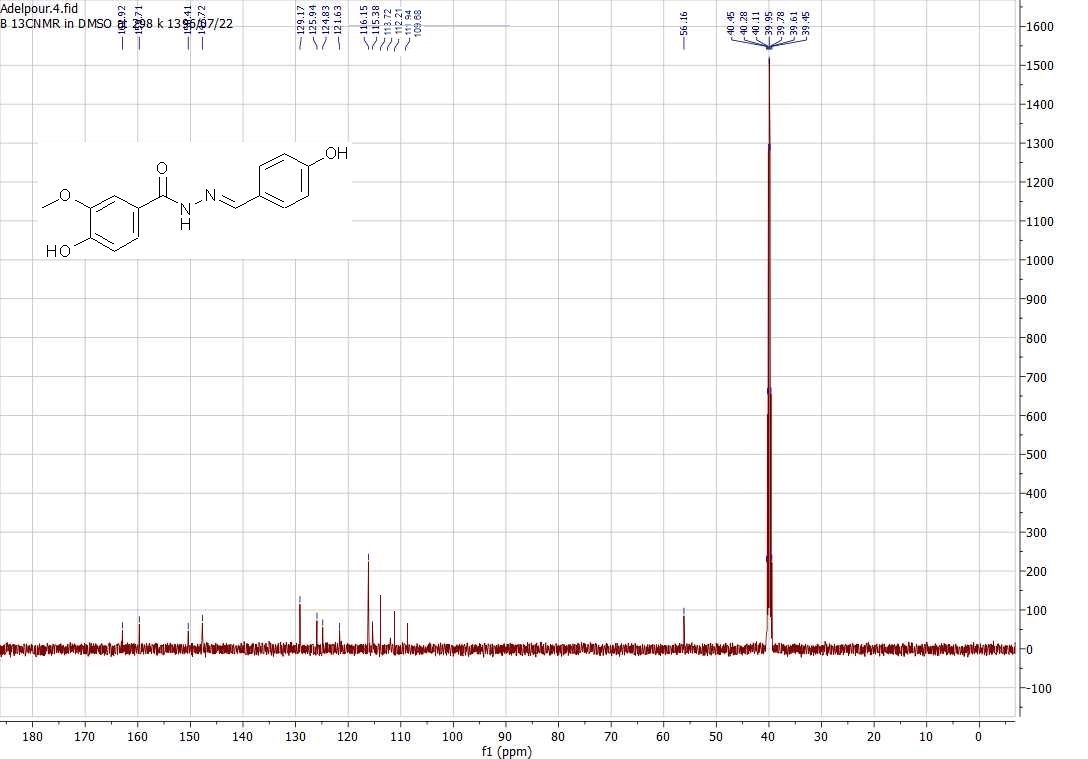
**

**Figure S8. IR of compound 4b**

**
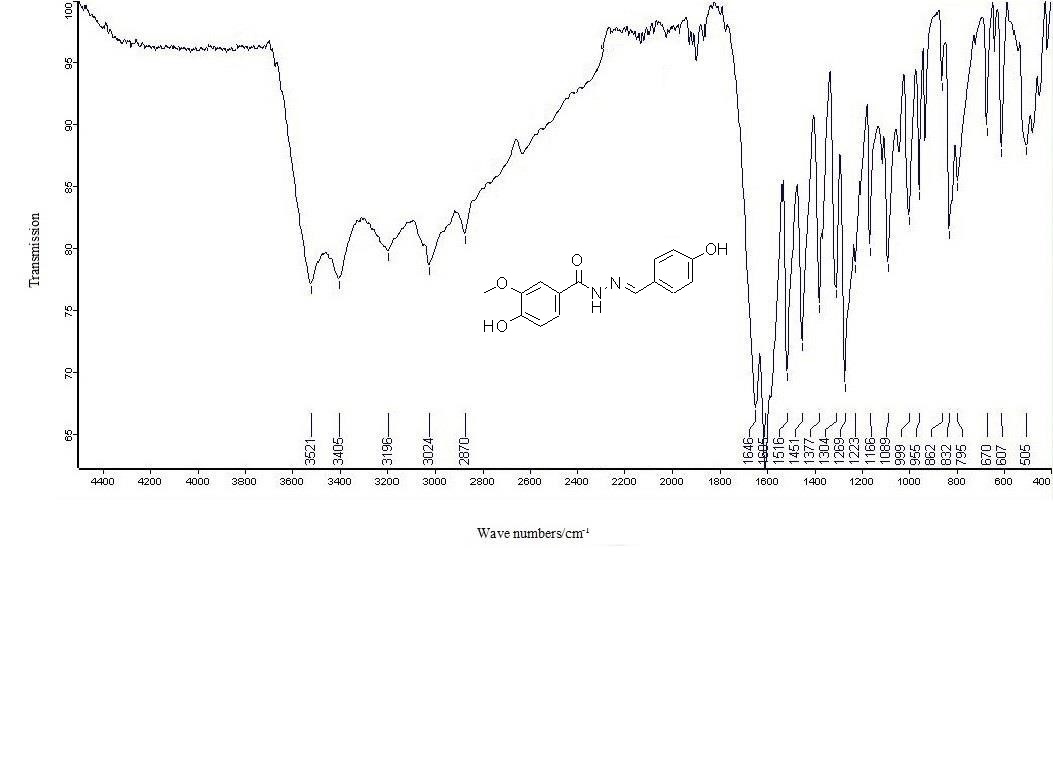
**

**Figure S9. Mass spectra of compound 4c**

**
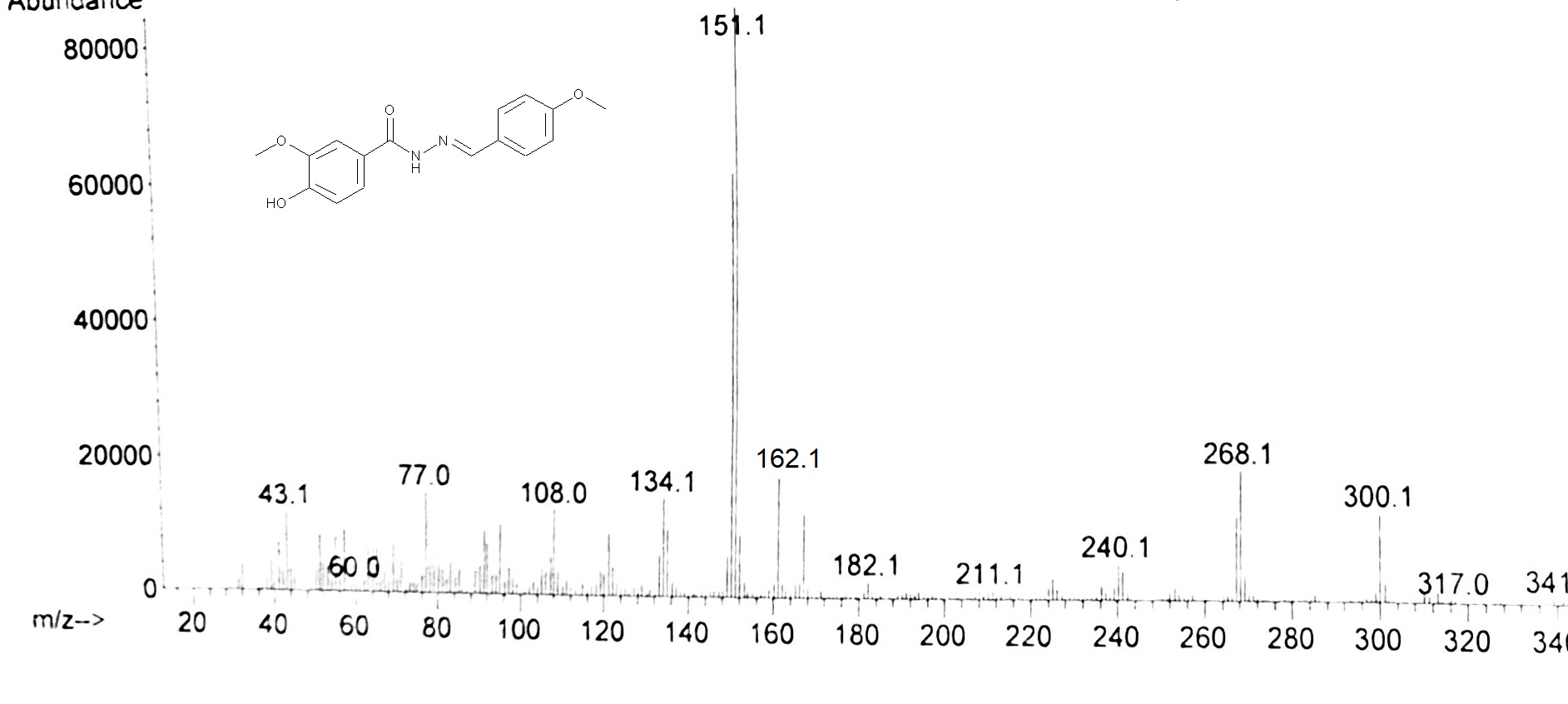
**

**Figure S10. ^1^H-NMR of compound 4c**

**
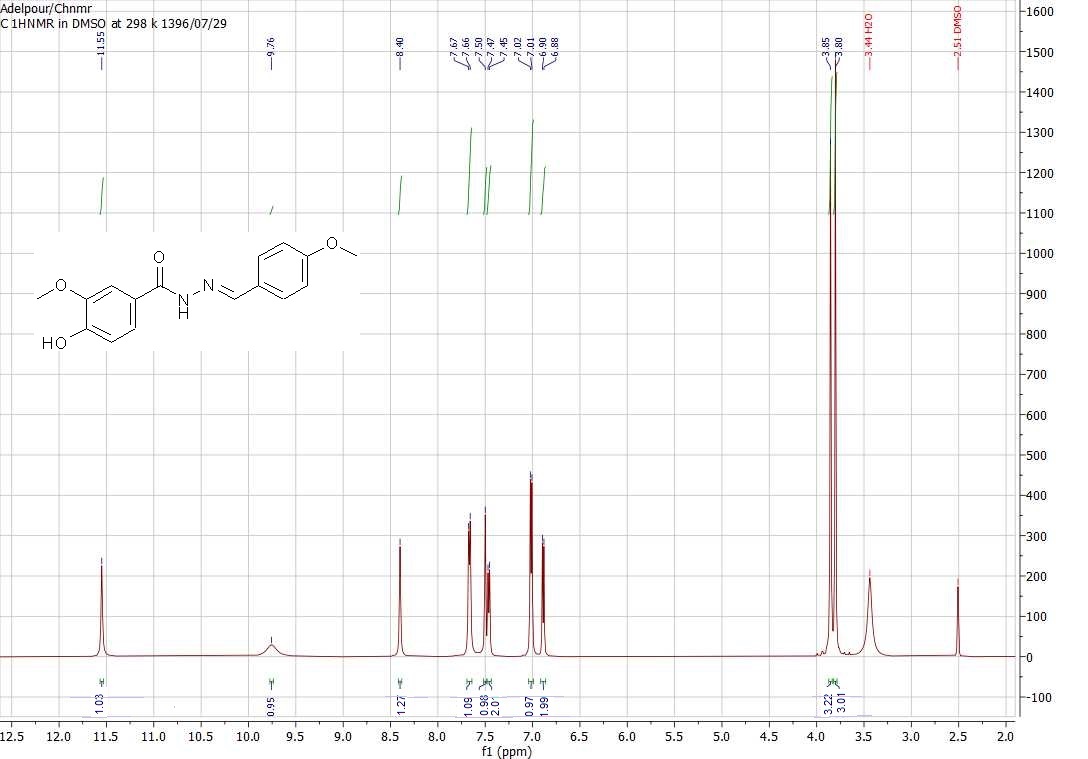
**

**Figure S11. ^13^C-NMR of compound 4c**

**
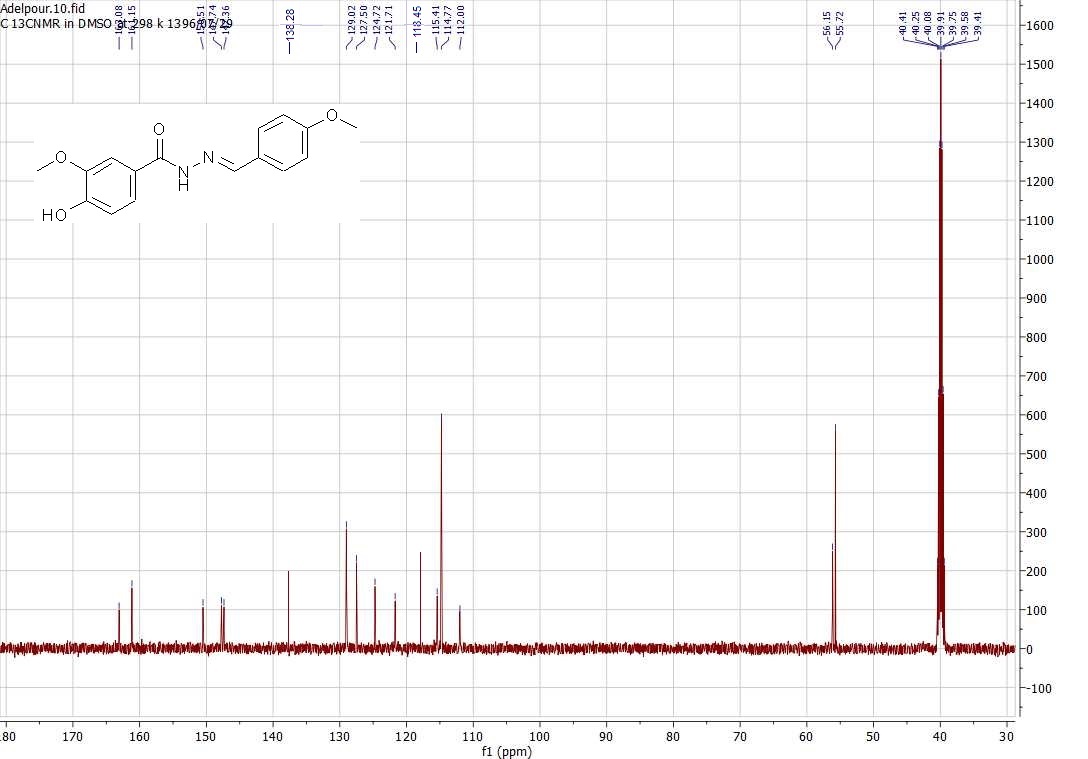
**

**Figure S12. IR of compound 4c**

**
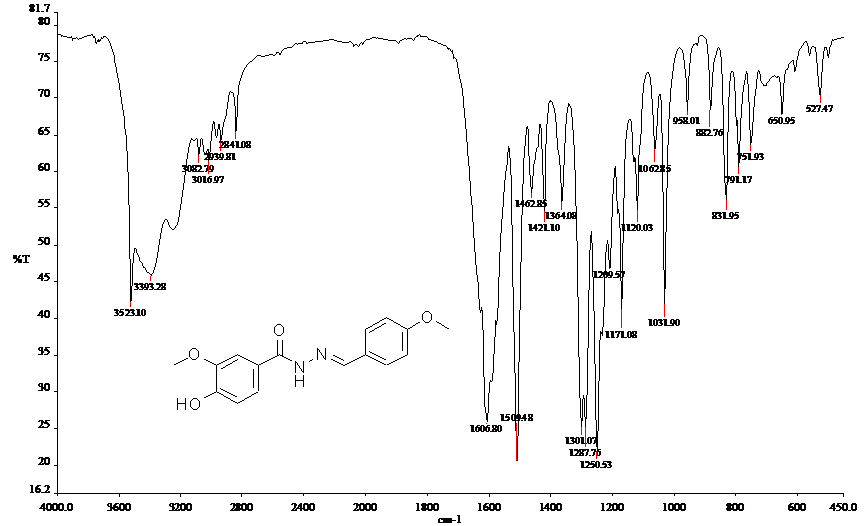
**

**Figure S13. Mass spectra of compound 4d**

**
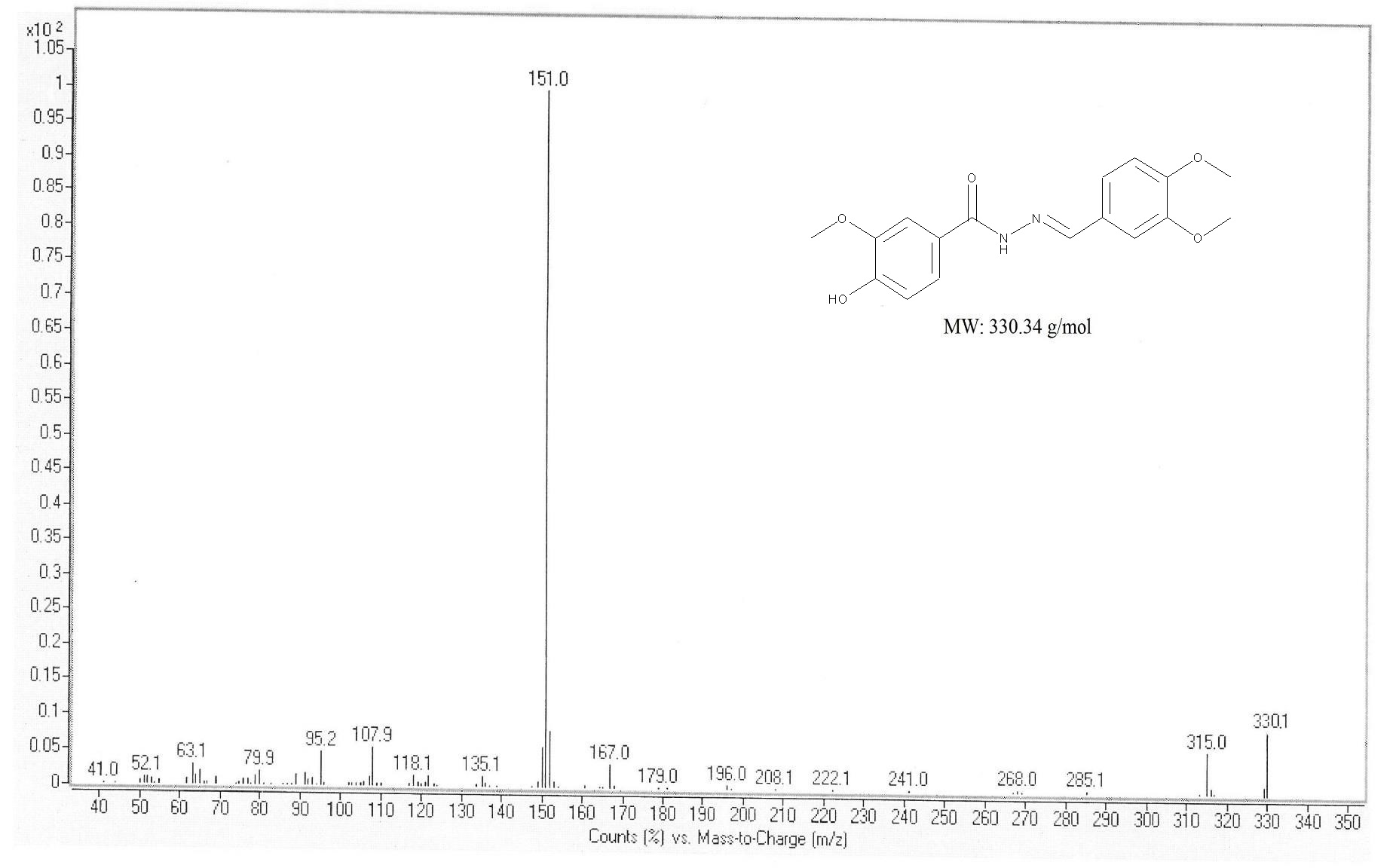
**

**Figure S14. ^1^H-NMR of compound 4d**

**
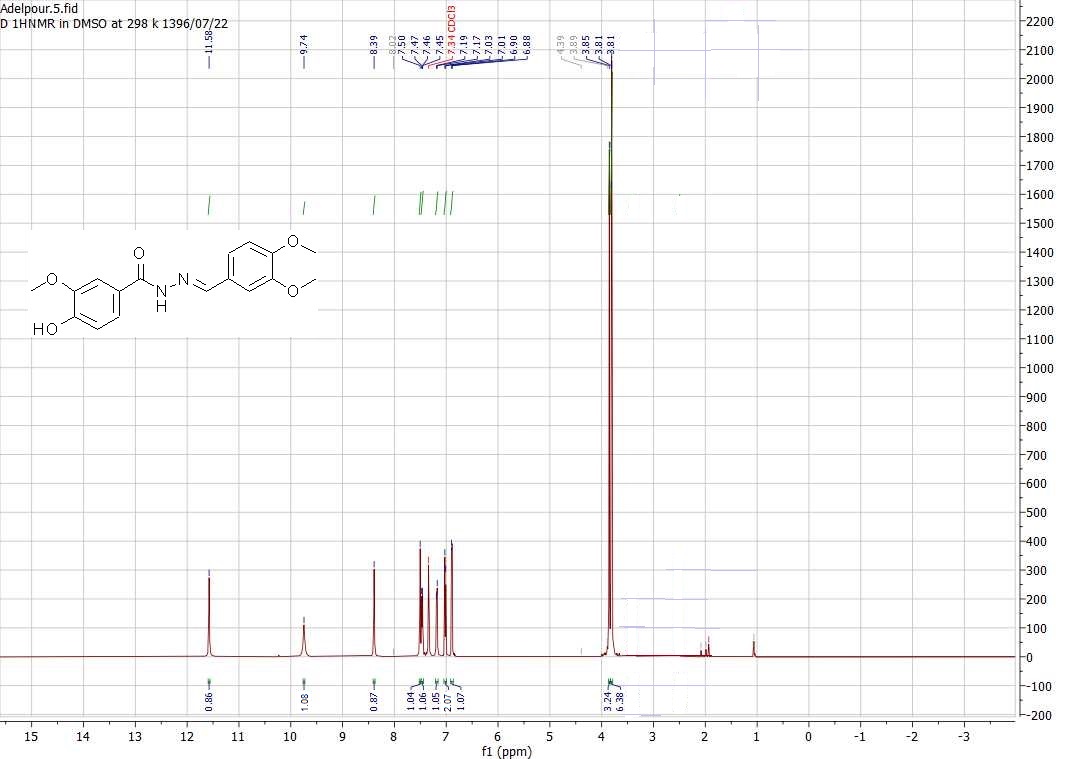
**

**Figure S15. ^13^C-NMR of compound 4d (expand)**

**
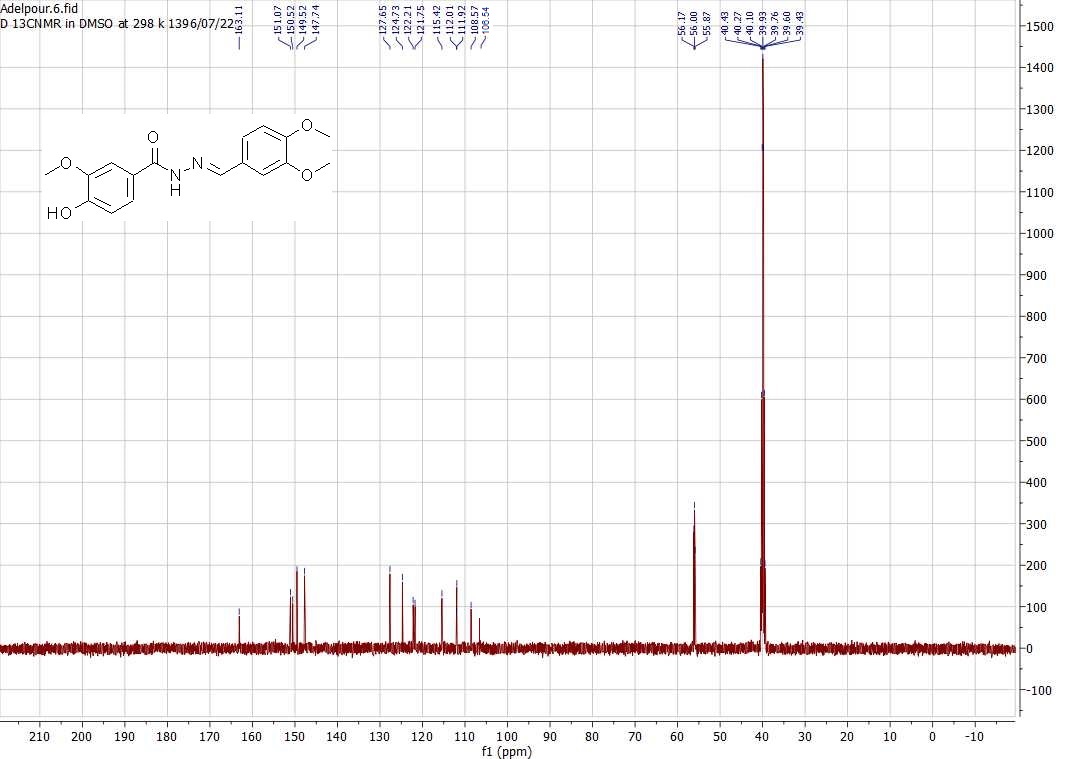
**

**Figure S16. IR of compound 4d**

**
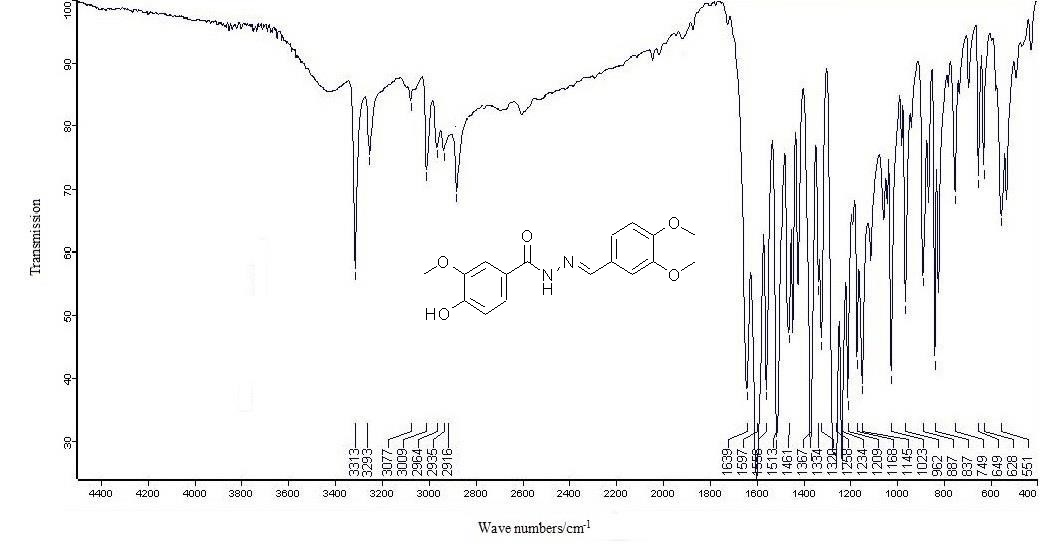
**

**Figure S17. Mass spectra of compound 4e**

**
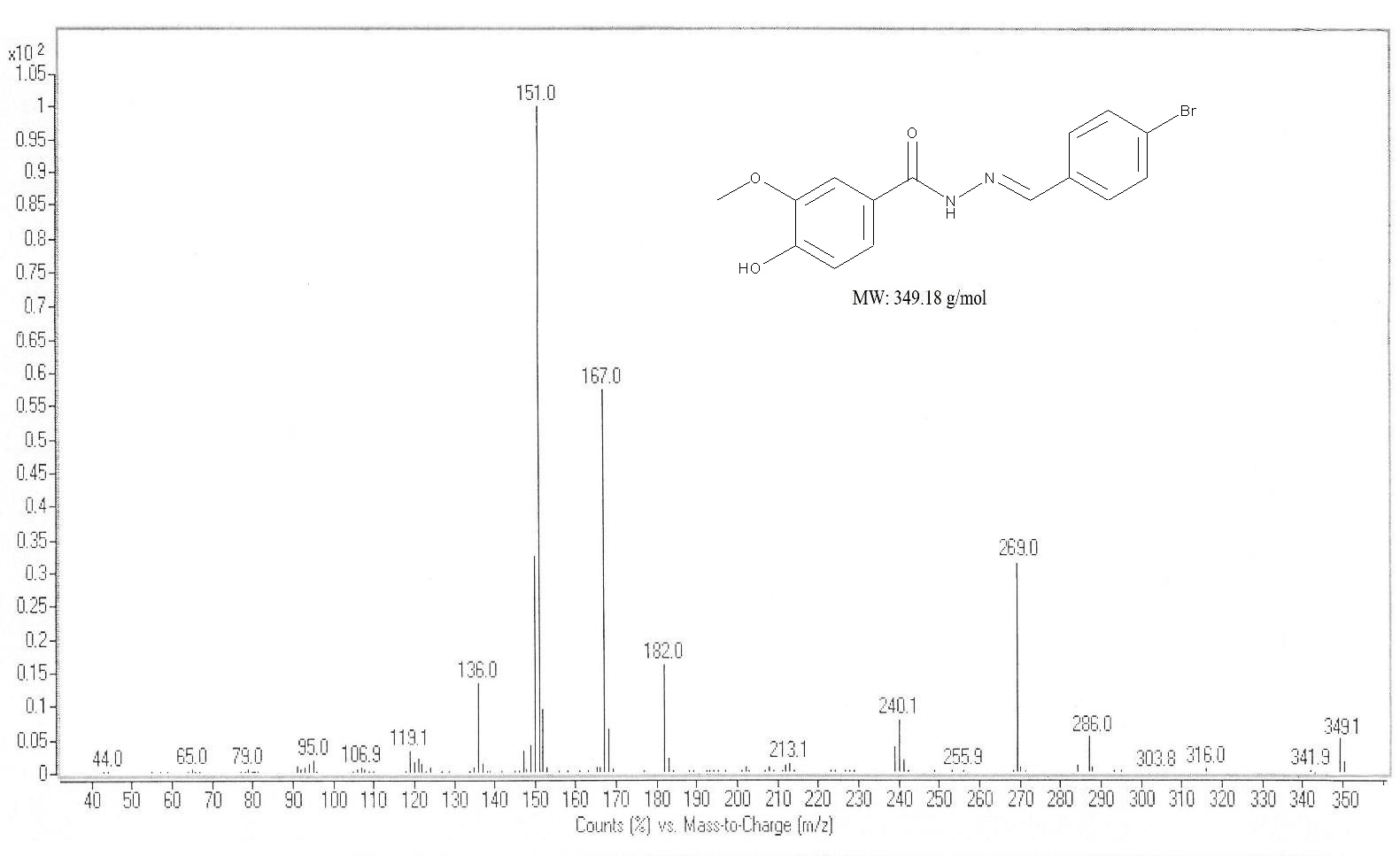
**

**Figure S18. ^1^H-NMR of compound 4e**


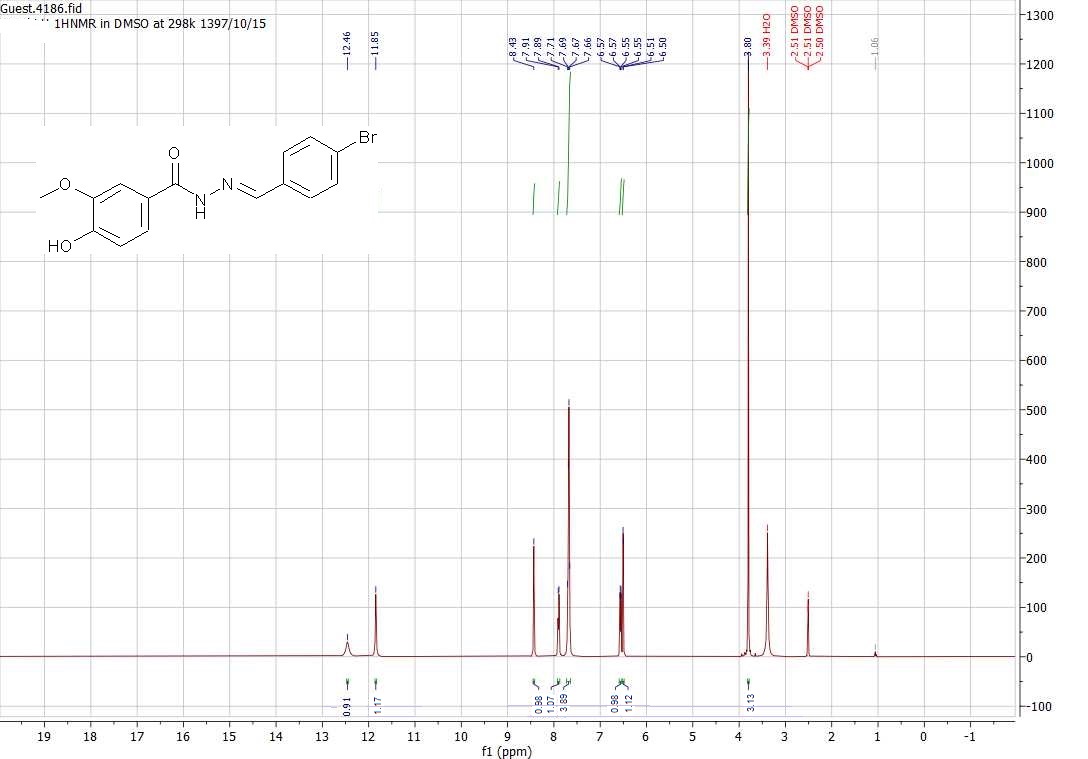


**Figure S19. ^13^C-NMR of compound 4e**

**
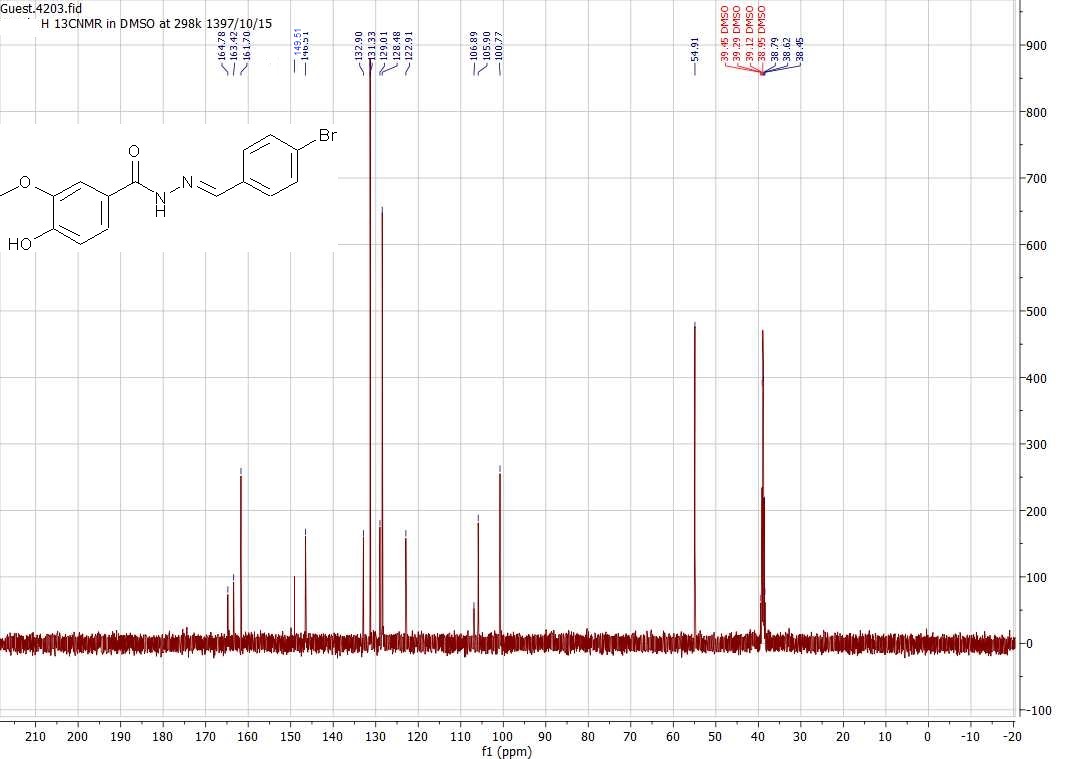
**

**Figure S20. IR of compound 4e**

**
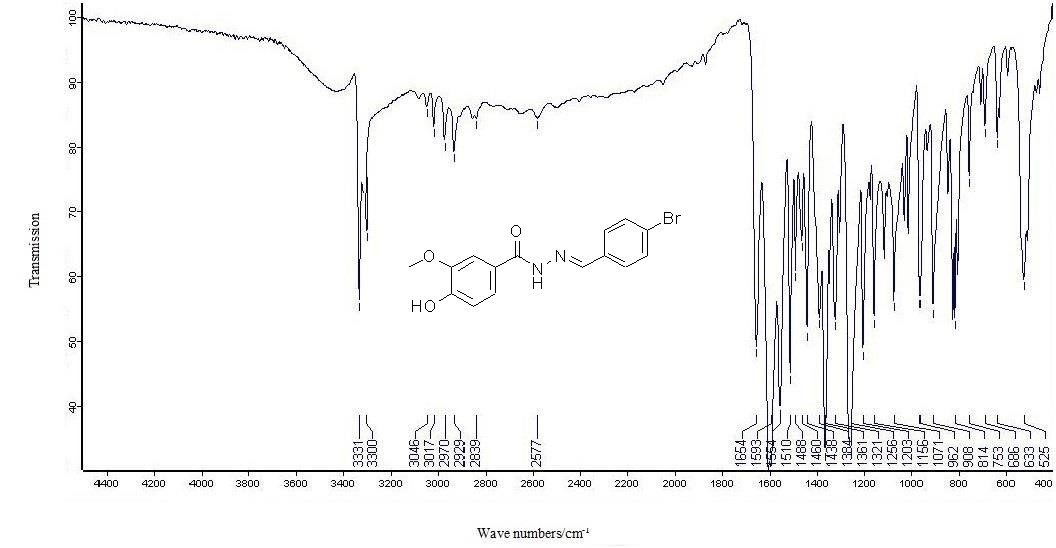
**

**Figure S21. Mass spectra of compound 4f**

**
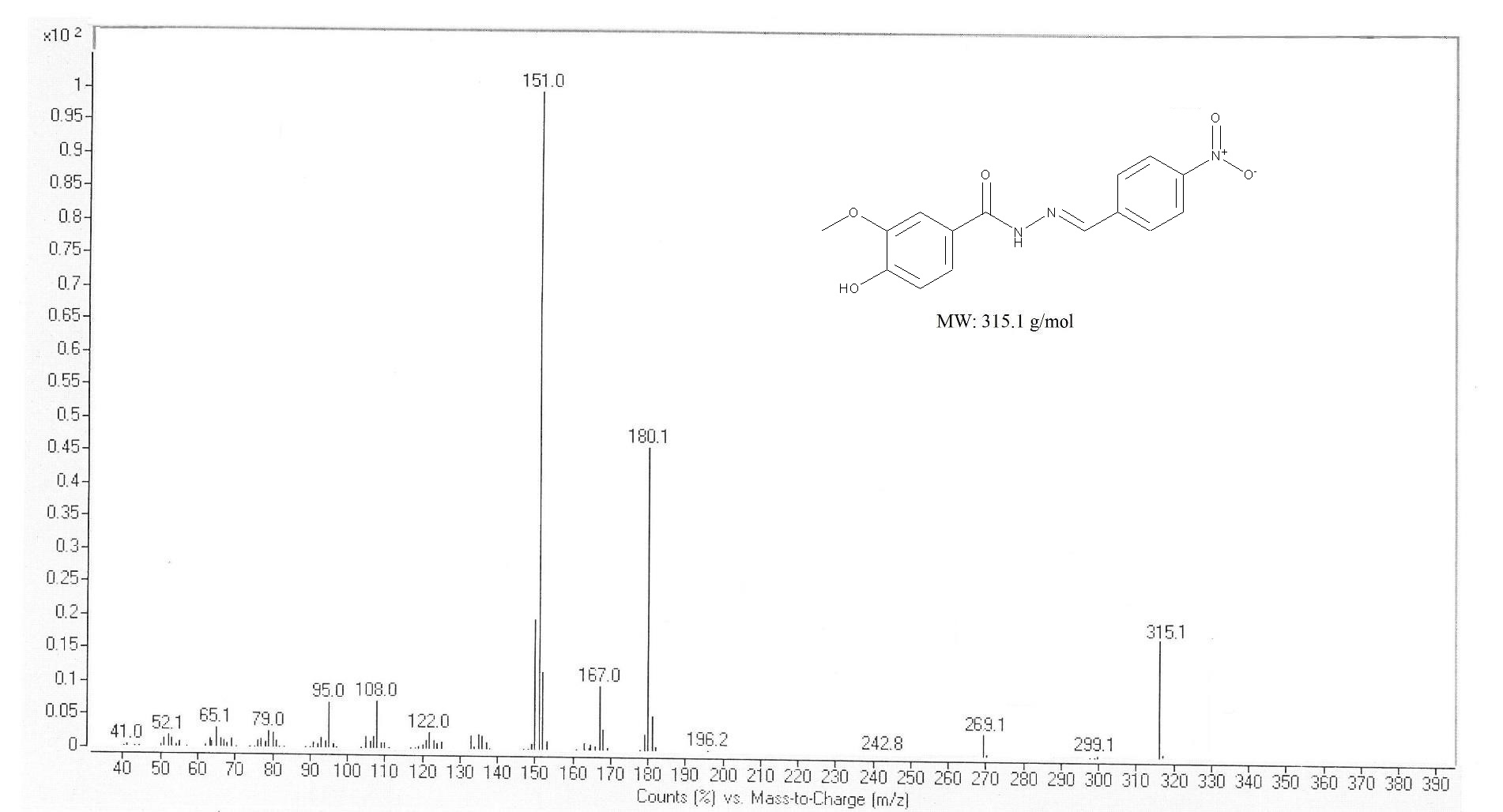
**

**Figure S22. ^1^H-NMR of compound 4f**

**
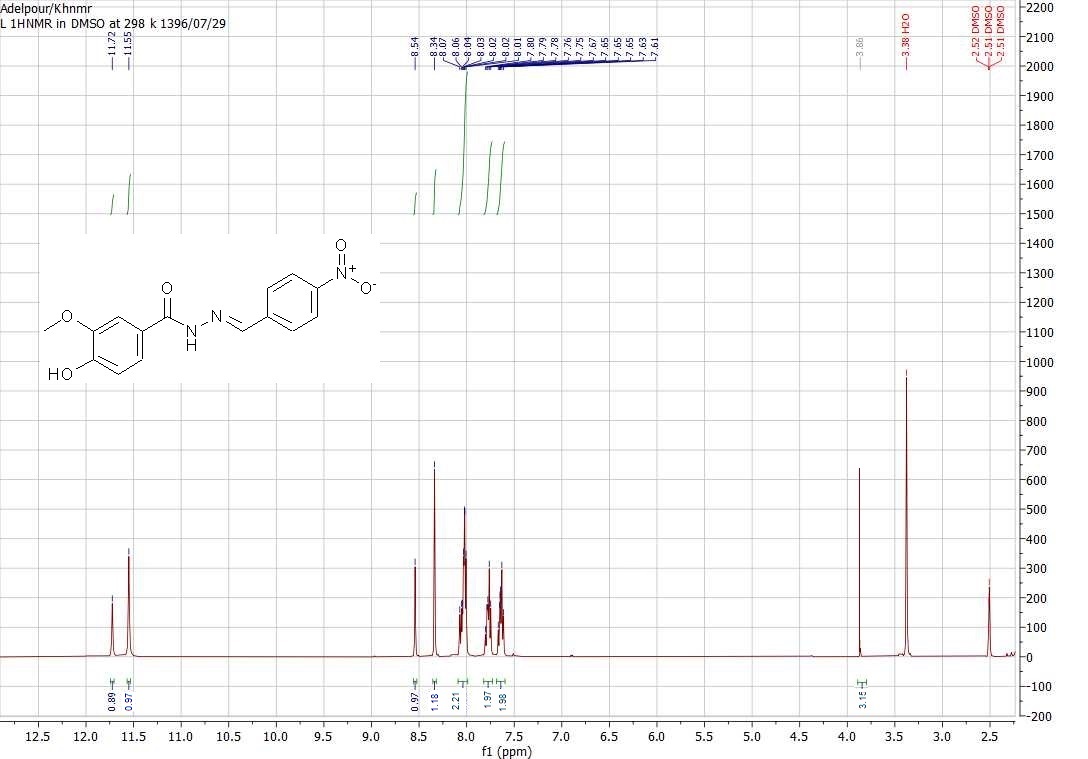
**

**Figure S23. ^13^C-NMR of compound 4f**

**
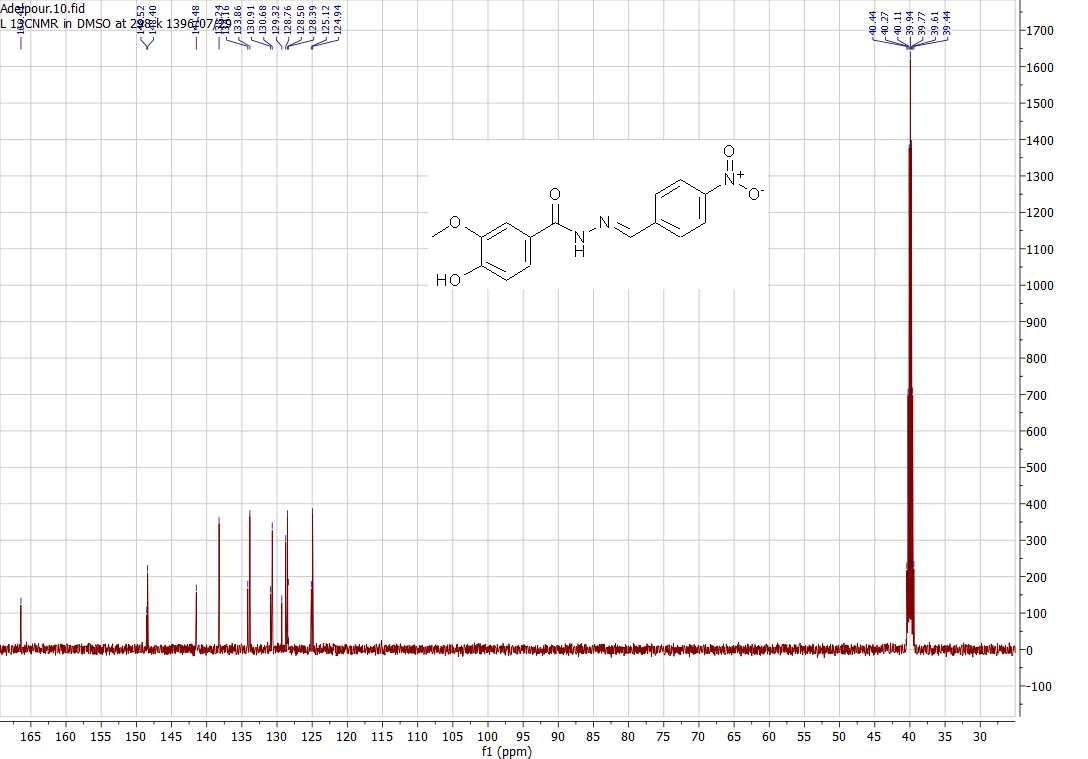
**

**Figure S24. IR of compound 4f**

**
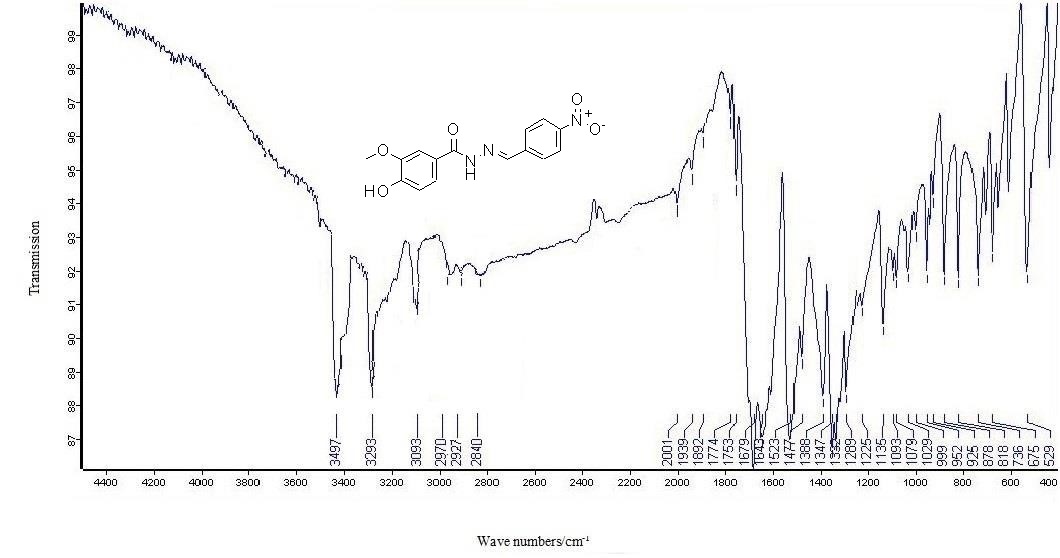
**

**Figure S25. Mass spectra of compound 4g**

**
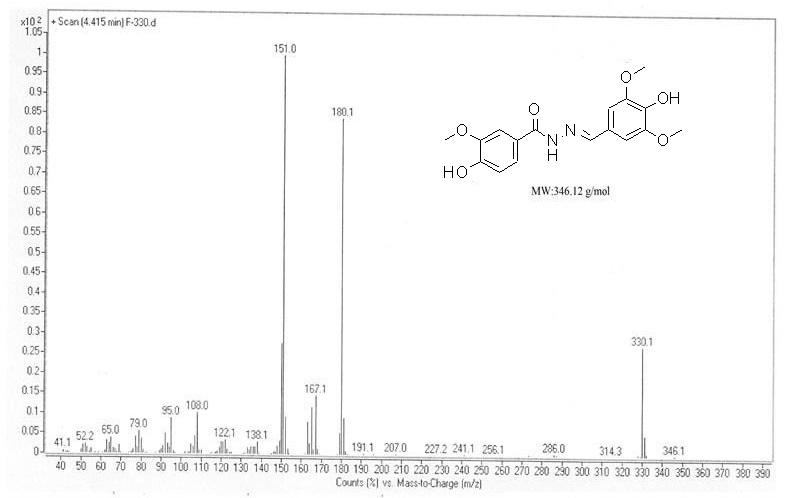
**

**Figure S26. ^1^H-NMR of compound 4g**

**
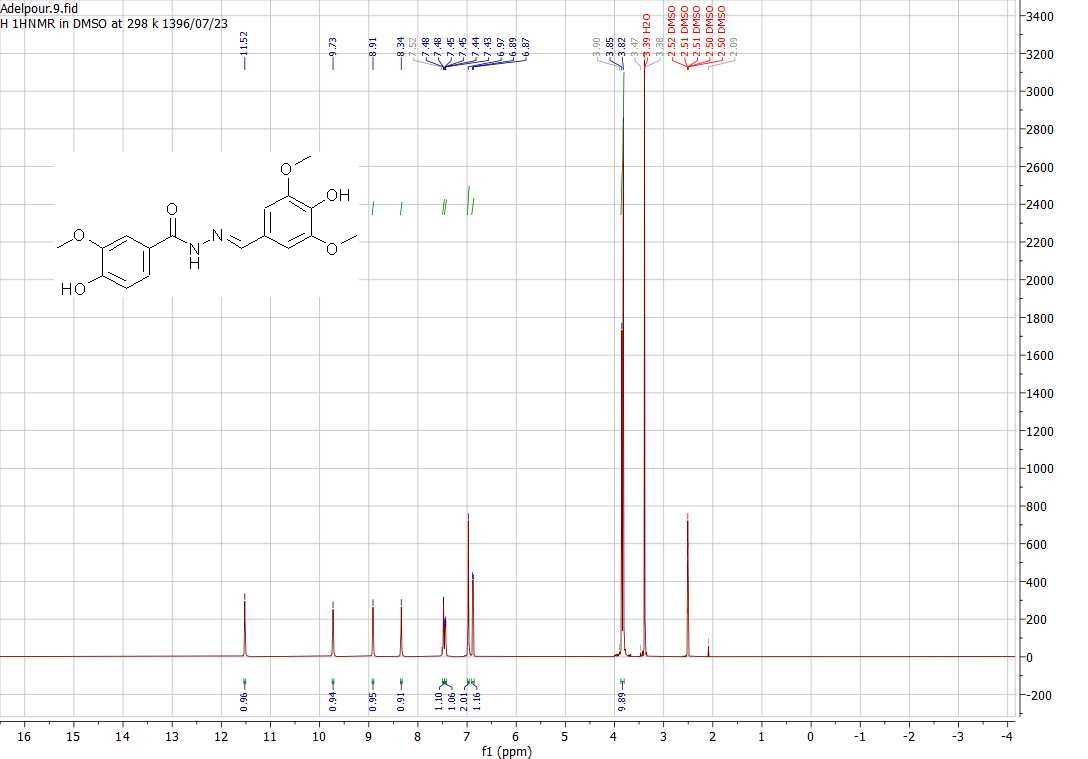
**

**Figure S27. ^13^C-NMR of compound 4g**

**
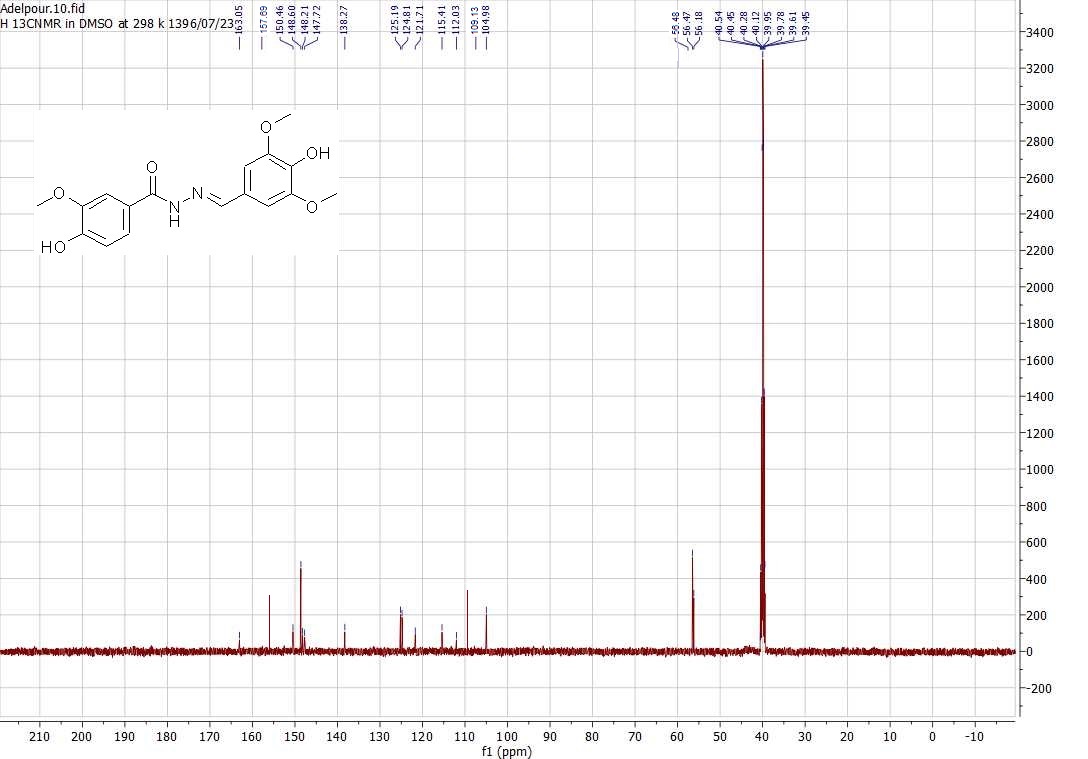
**

**Figure S28. IR of compound 4g**

**
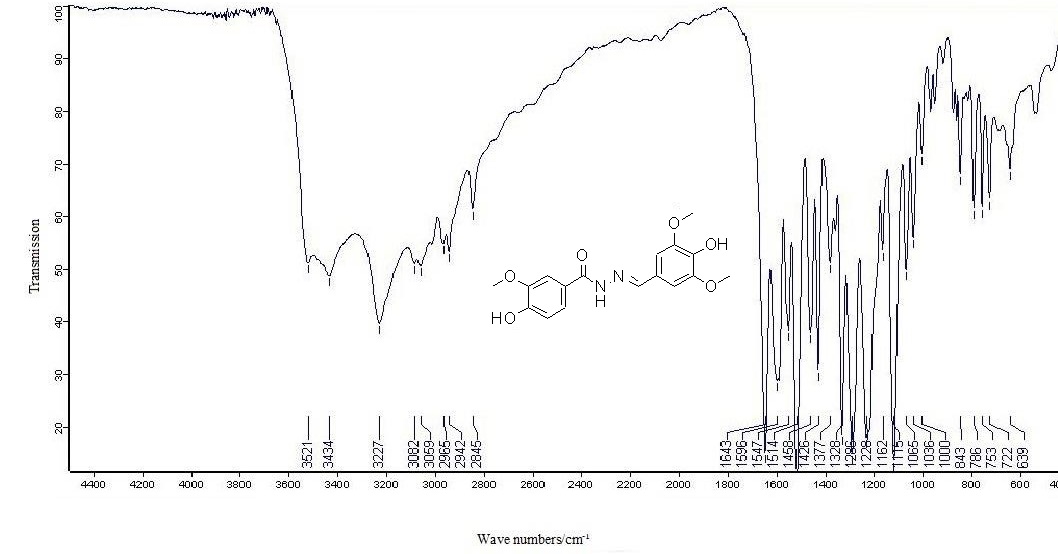
**

**Figure S29. Mass spectra of compound 4h**

**
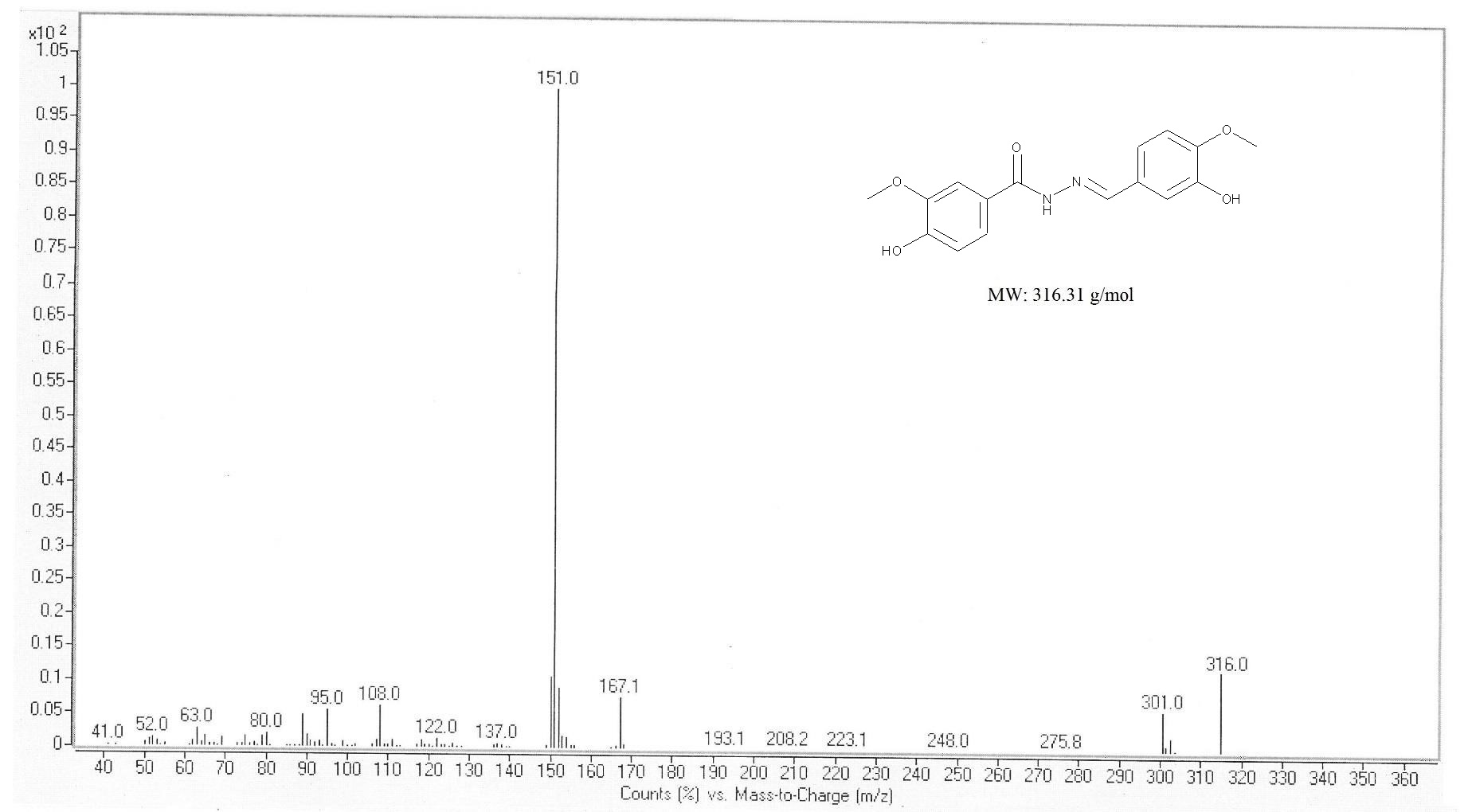
**

**Figure S30. ^1^H-NMR of compound 4h**

**
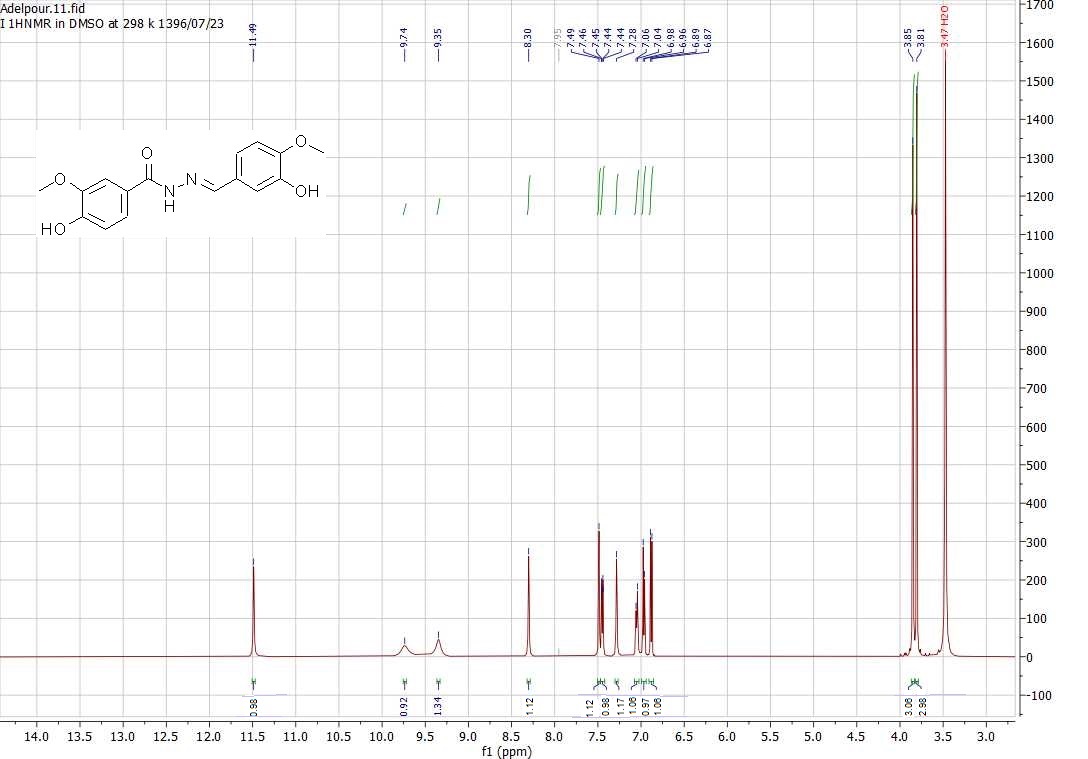
**

**Figure S31. ^13^C-NMR of compound 4h**

**
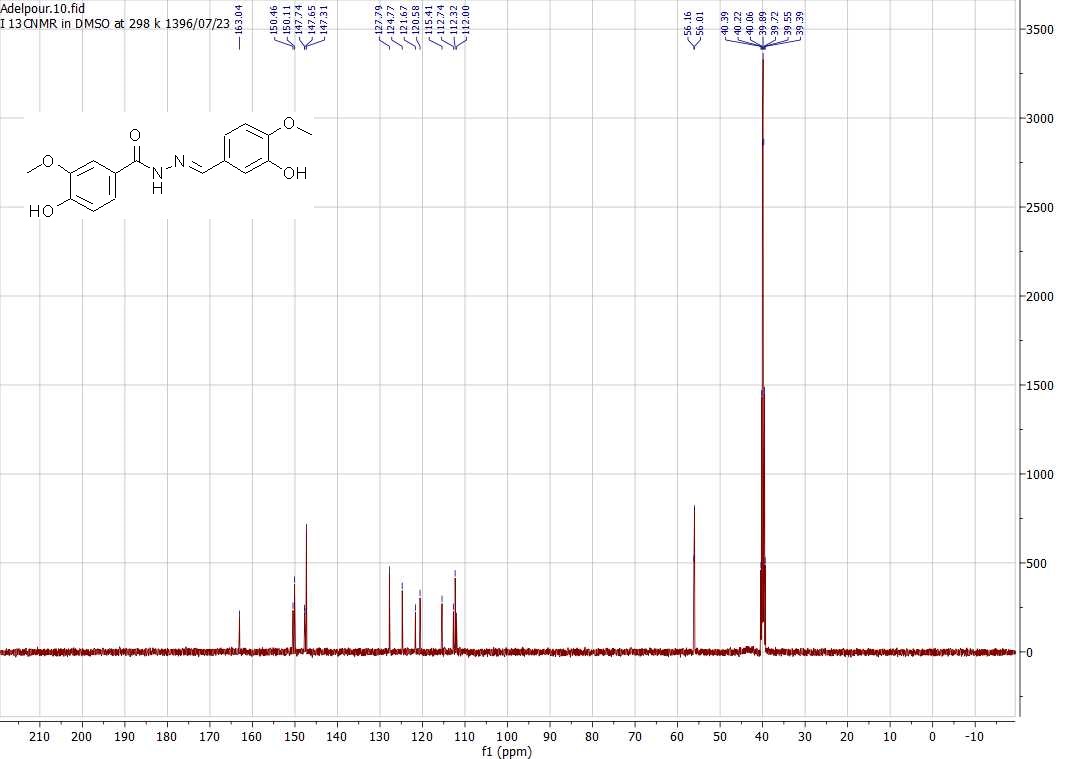
**

**Figure S32. IR of compound 4h**

**
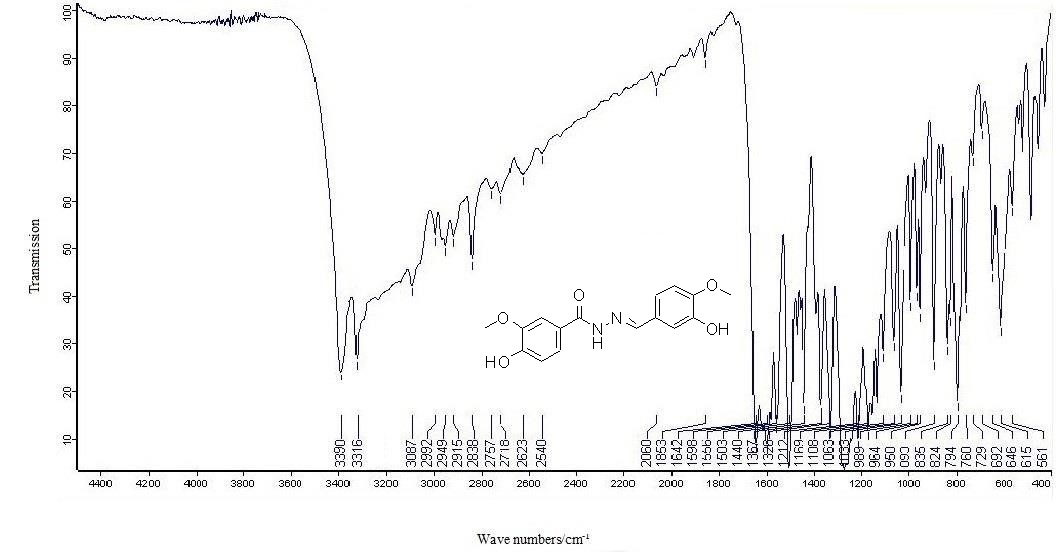
**

**Figure S33. Mass spectra of compound 4i**

**
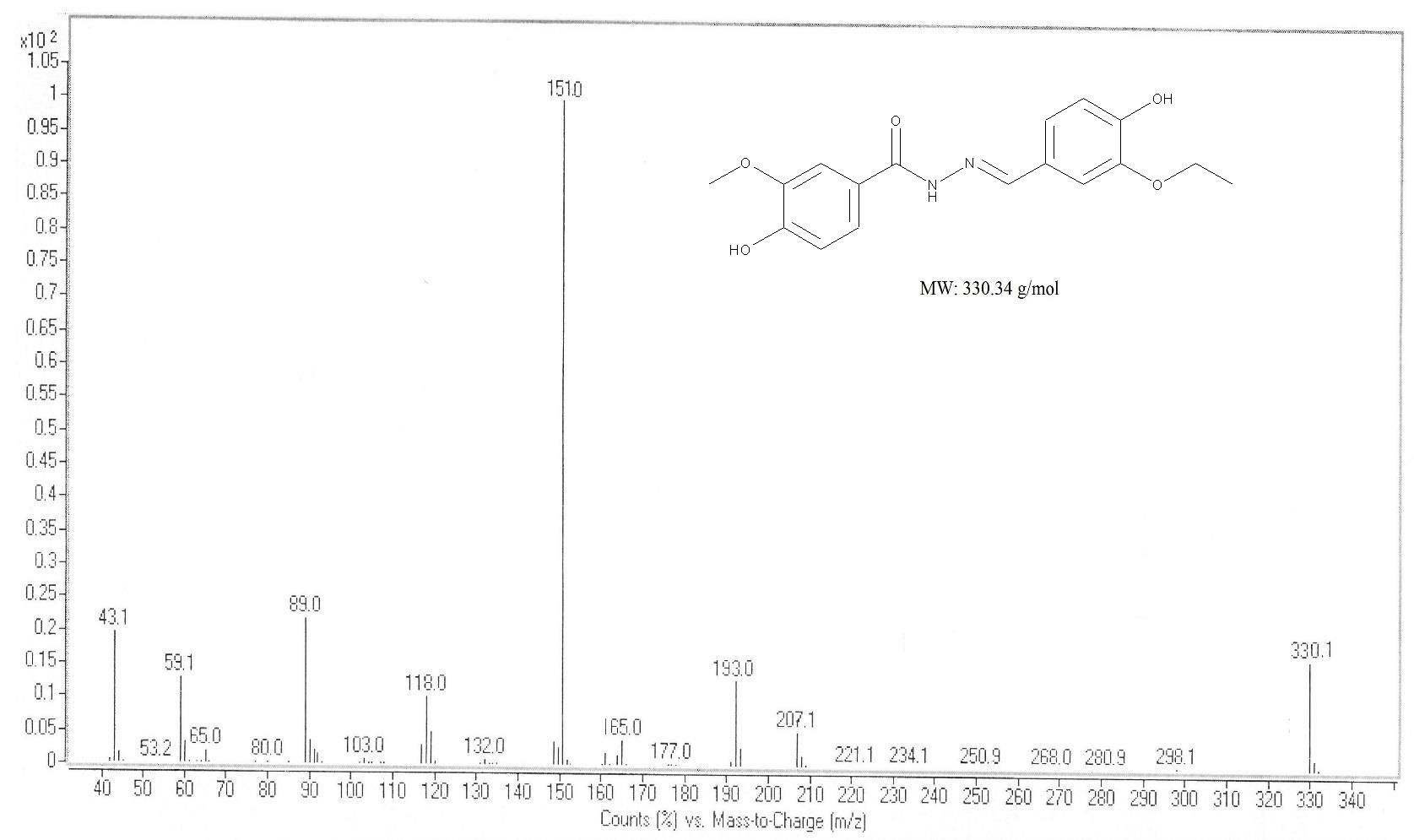
**

**Figure S34. ^1^H-NMR** **of compound 4i**

**
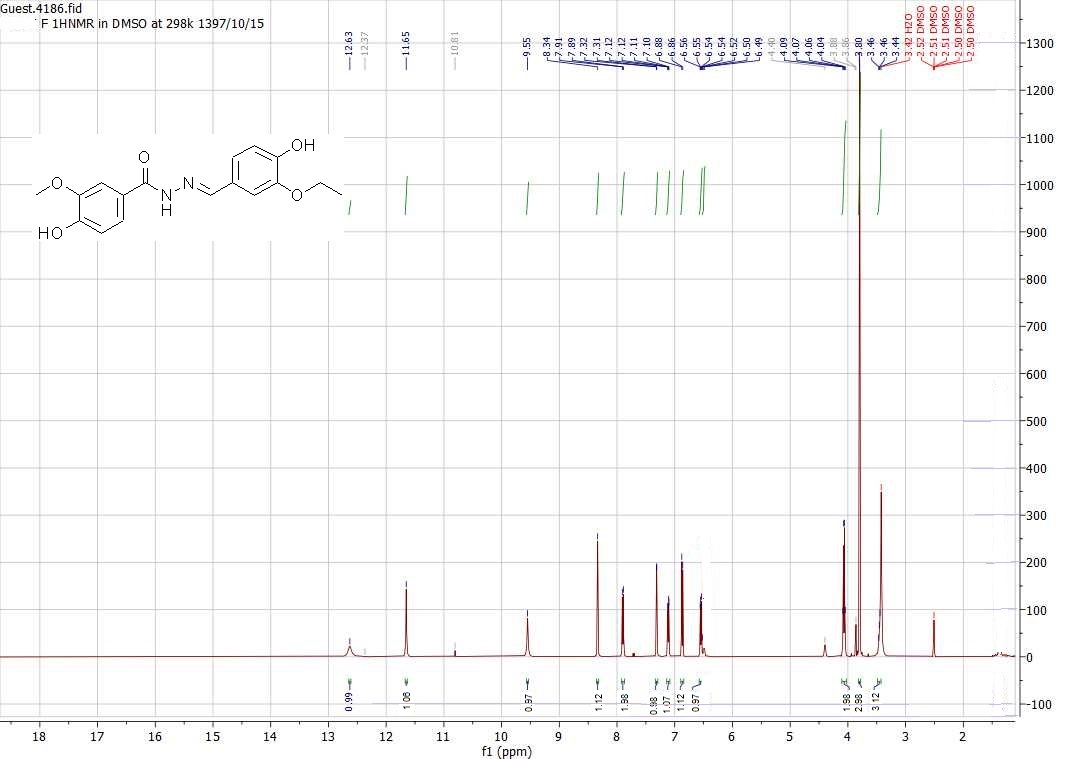
**

**Figure S35. ^13^C-NMR** **of compound 4i**

**
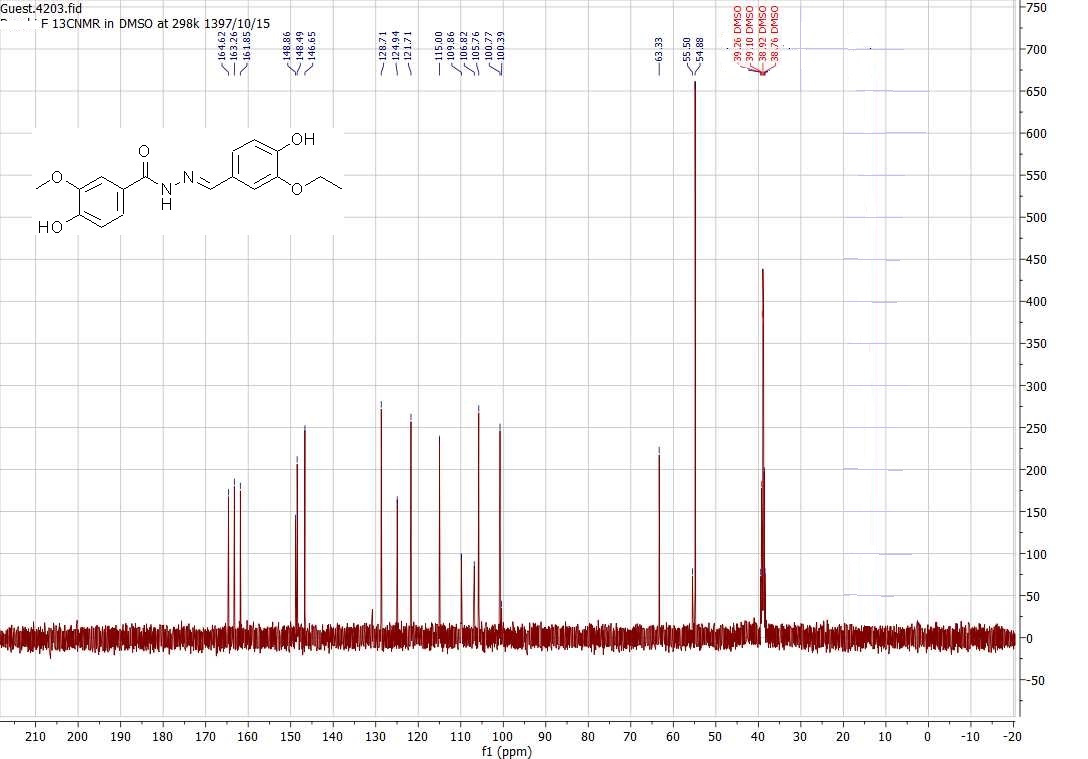
**

**Figure S36. IR** **of compound 4i**

**
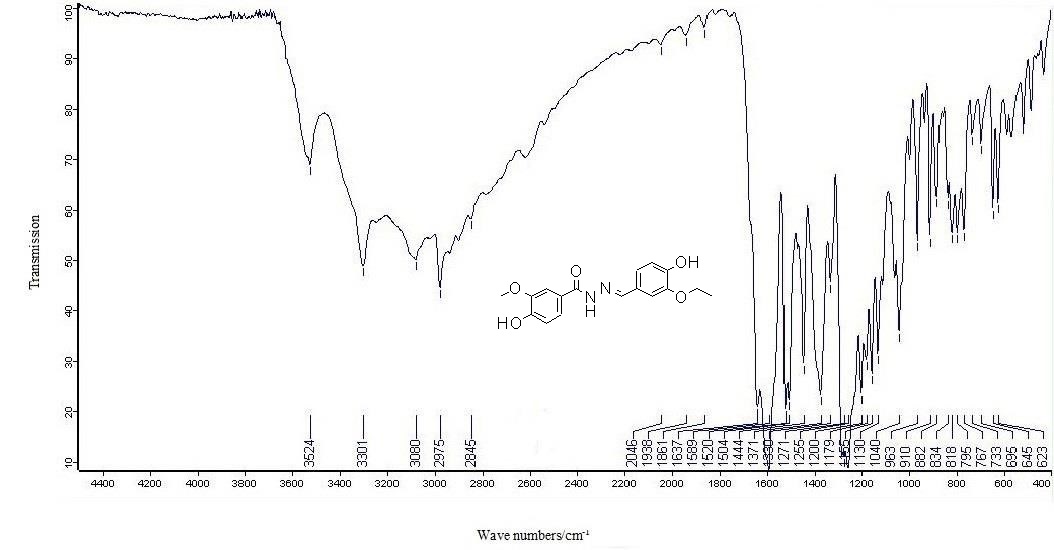
**

**Figure S37. Mass** **of compound 4j**

**
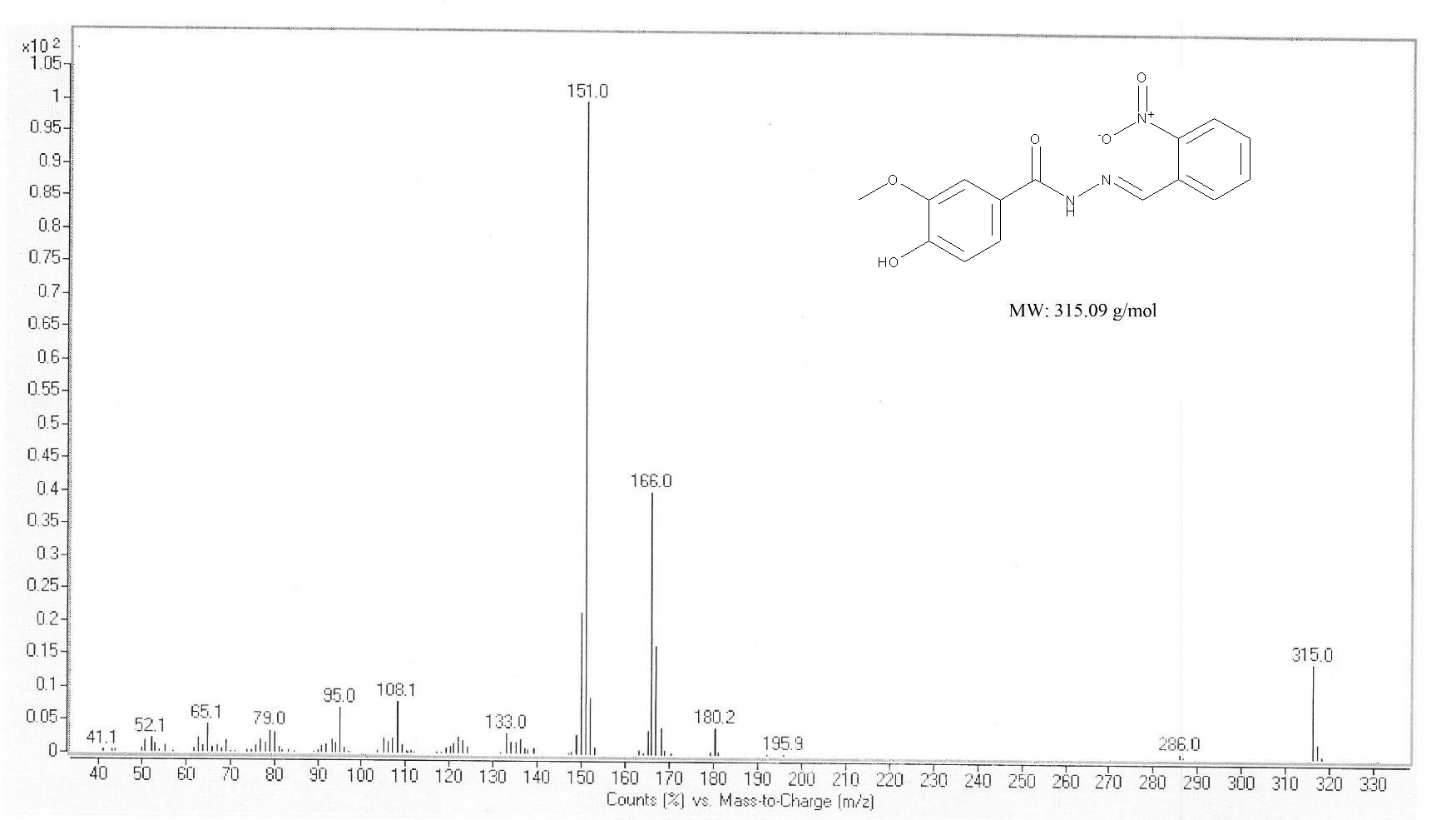
**

**Figure S38. ^13^H-NMR** **of compound 4j**

**
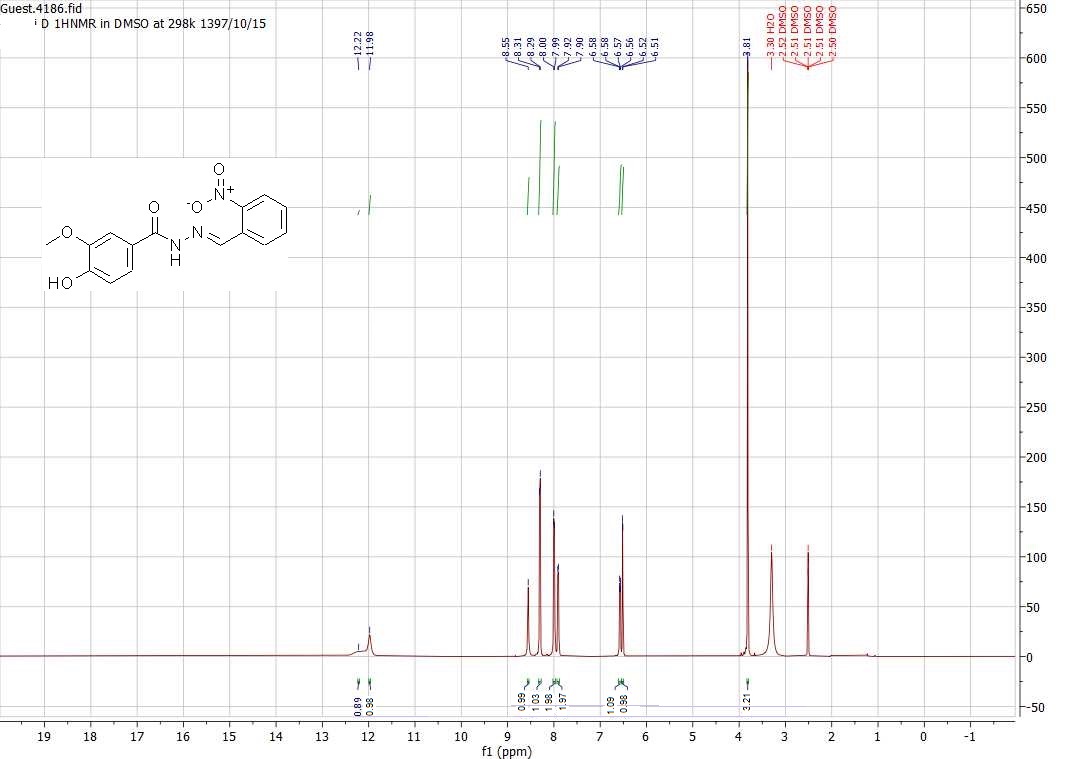
**

**Figure S39. ^13^C-NMR** **of compound 4j**

**
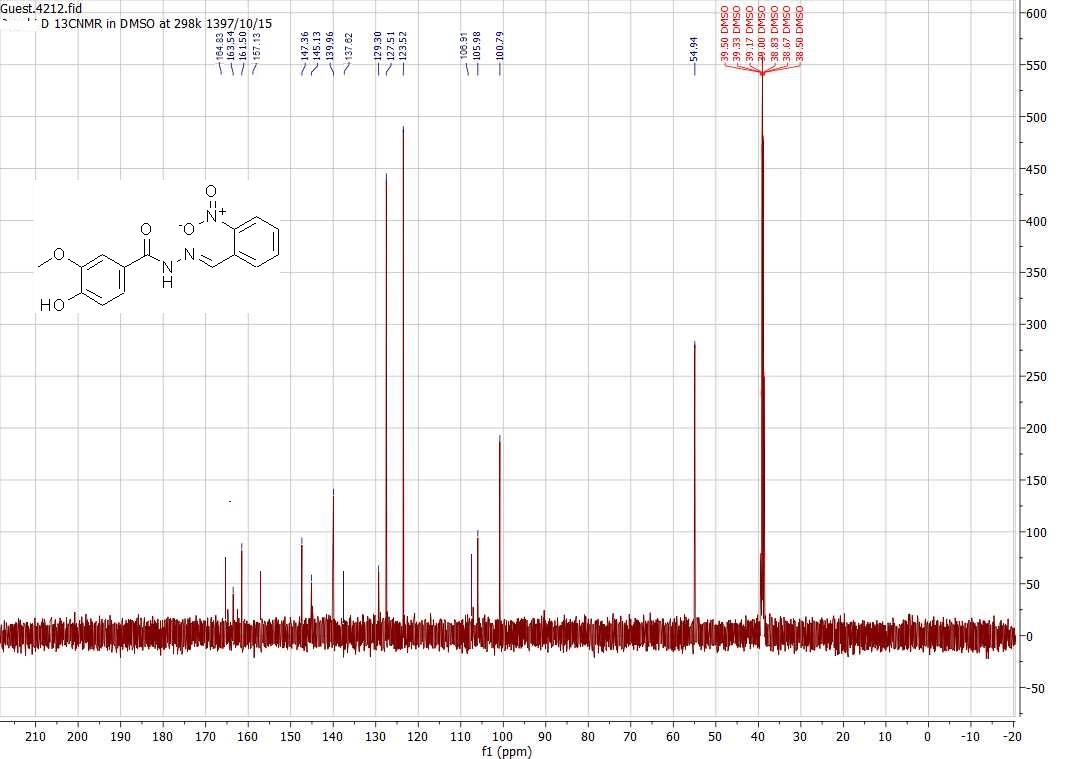
**

**Figure S40. IR** **of compound 4j**

**
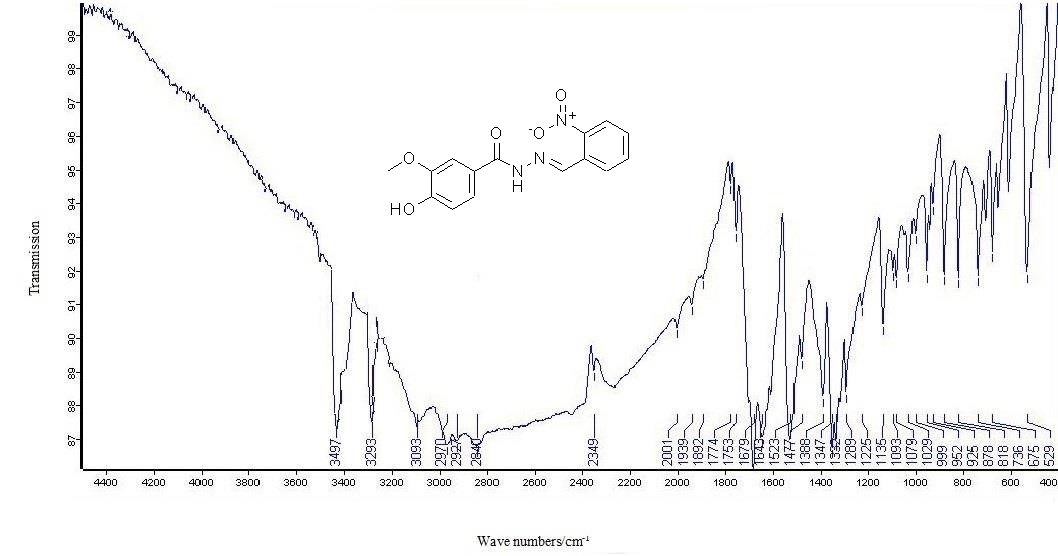
**

**Figure S41. Mass** **of compound 4k**

**
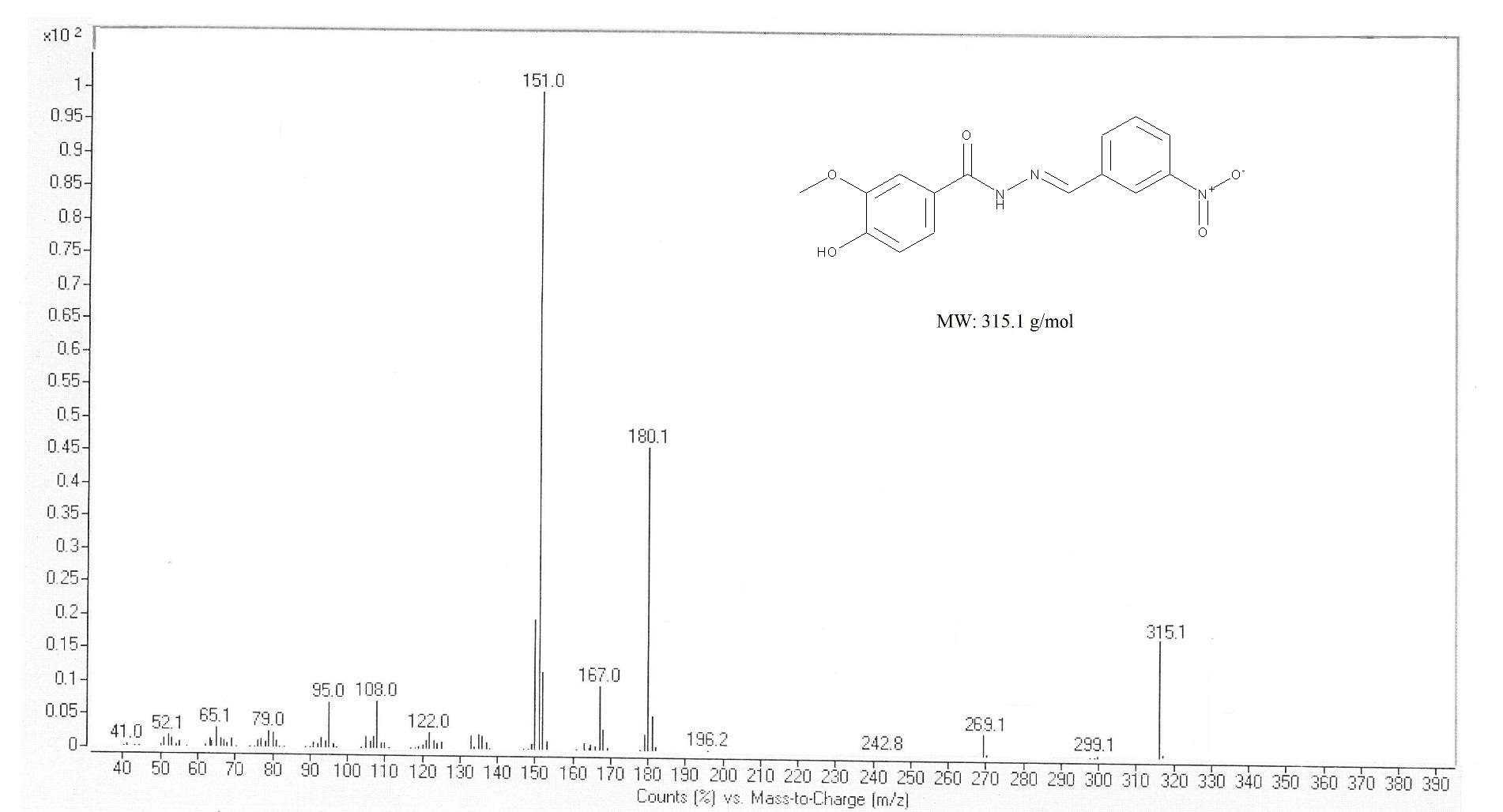
**

**Figure S42. ^1^H-NMR** **of compound 4k**

**
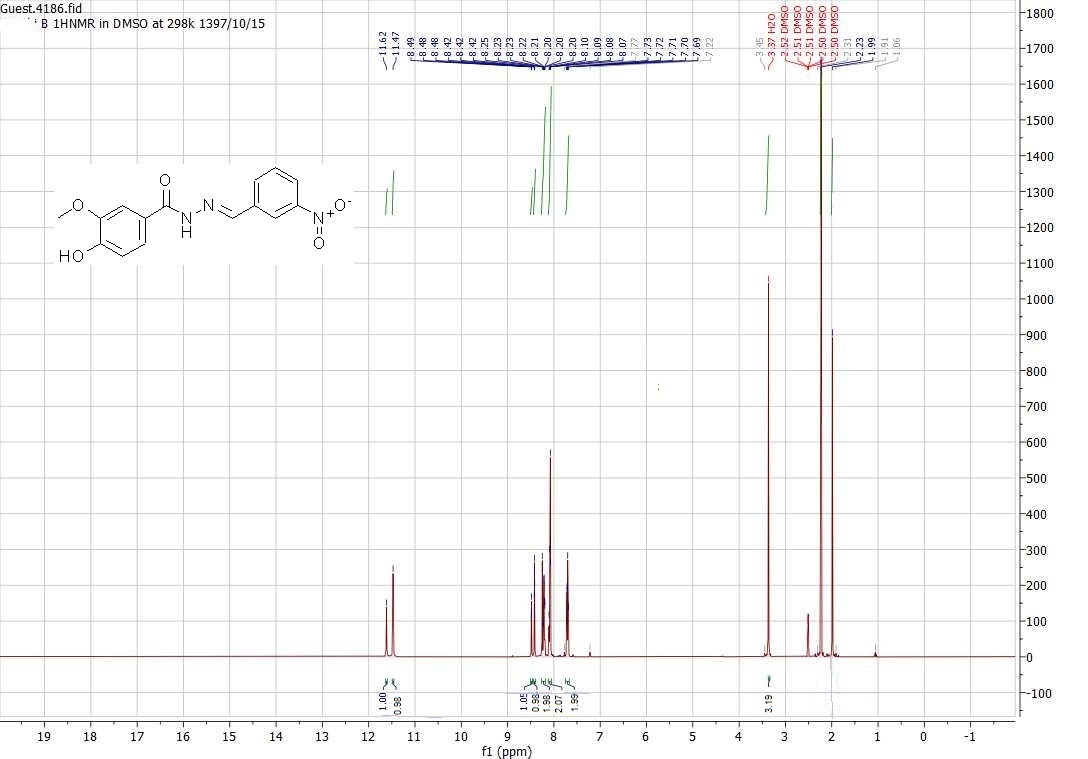
**

**Figure S43. ^13^C-NMR** **of compound 4k**

**
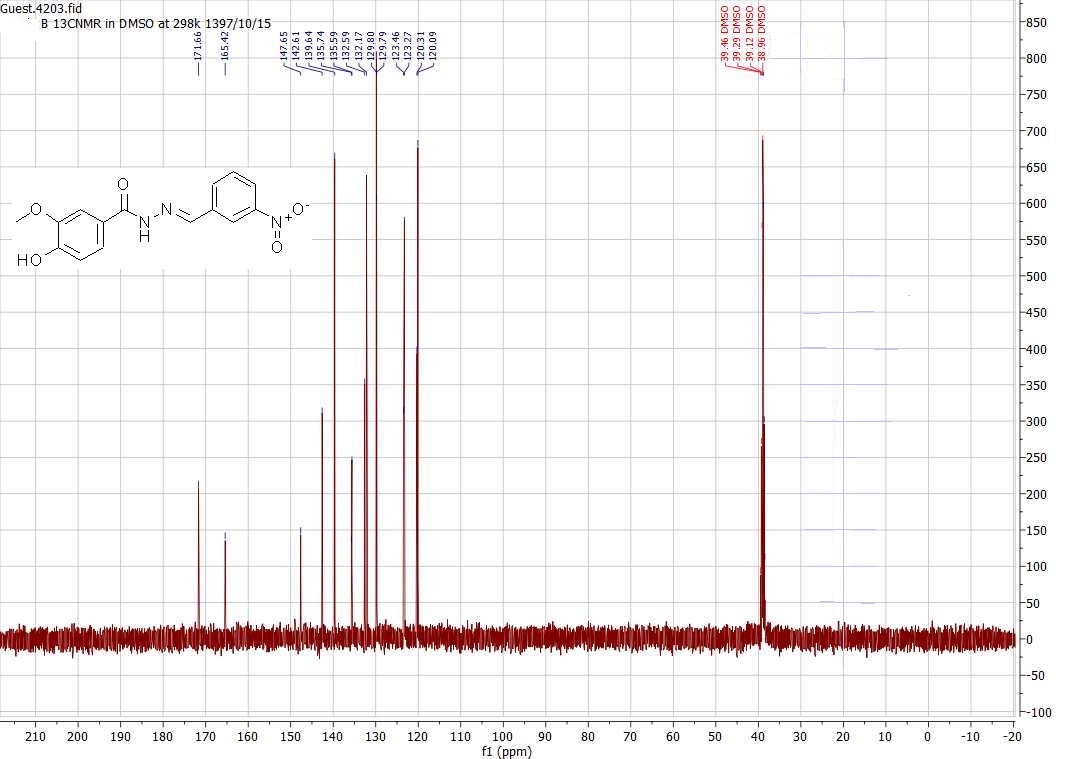
**

**Figure S44. IR** **of compound 4k**

**
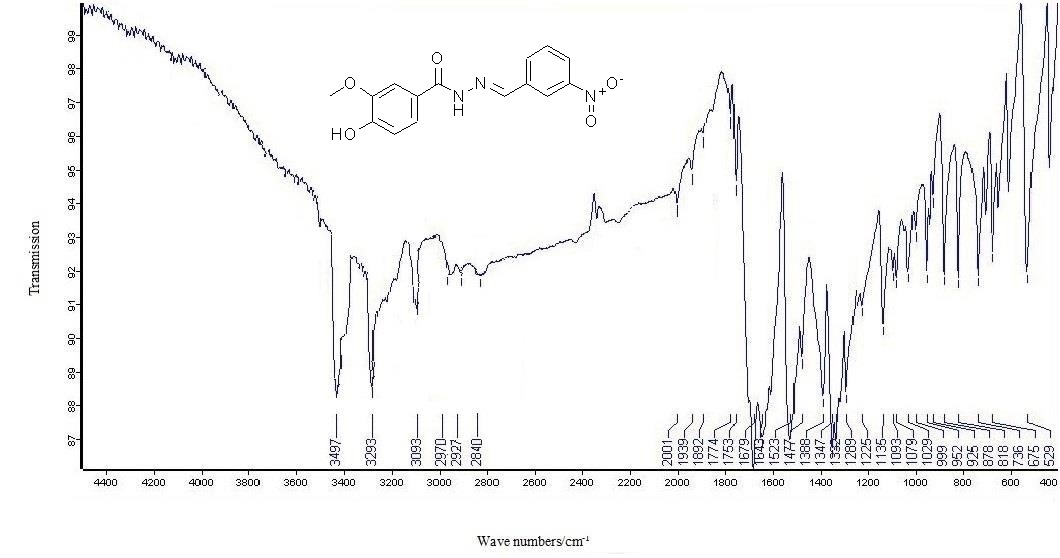
**
